# Hypoxia Associated Integration of Epigenetic, Metabolic, and Immune Biomarkers in Blood and Urine for Early Colorectal Cancer Detection: A Multimarker Panel

**DOI:** 10.3390/diagnostics16121753

**Published:** 2026-06-06

**Authors:** Christopher Birigwa, Bing Qu, Yongqing Tong, Teng Zuo, Wenzheng Yuan, Qingbo Wang, Wei Song, Weiwei Wan, Jing Xiong, Jianfei Luo, Qiang Tong

**Affiliations:** 1 Department of Gastrointestinal Surgery, Renmin Hospital of Wuhan University, Wuhan 430060, China; birigwachristopherdominic@gmail.com (C.B.); qbwangkk@163.com (Q.W.); whdxsw@whu.edu.cn (W.S.); wanww1982@163.com (W.W.); qiangtong@whu.edu.cn (Q.T.); 2 Department of Clinical Laboratory, Renmin Hospital of Wuhan University, Wuhan 430060, China; tytsing@whu.edu.cn; 3 School of Medicine, Wuhan University of Science and Technology, Wuhan 430065, China; xiongjing@wust.edu.cn

**Keywords:** colorectal cancer, liquid biopsy, methylated SEPT9 (mSEPT9), N^1^,N^12^-diacetylspermine (DiAcSpm), multimarker diagnostic panel, circulating cell-free DNA (cfDNA), inflammatory biomarkers (NLR, PLR, LMR), non-invasive cancer detection, diagnostic accuracy

## Abstract

**Background:** Detection of colorectal cancer (CRC) currently relies mainly on invasive procedures such as colonoscopy. In experimental models, tumor hypoxia induces epigenetic, metabolic, and immune changes via hypoxia-inducible factor (HIF) signaling. Building on these published insights, this study evaluated whether a panel of biomarkers previously associated with hypoxia-related processes, plasma methylated SEPT9 (mSEPT9), urinary N^1^, N^12^-diacetylspermine (DiAcSpm), and systemic inflammatory indices (NLR, PLR, and LMR)—could be combined into a non-invasive diagnostic panel and compared with standard serum tumor markers. This study focused solely on diagnostic performance; it did not directly assess tumor hypoxia or HIF expression in patients. This study was conducted in a clinical diagnostic setting (patients with confirmed CRC, polyps, or benign surgical conditions) and does not represent a population-based screening cohort. Limitations include the lack of an external validation cohort; all analyses were performed on a single dataset, and the reported performance metrics may be optimistic. Independent validation is required before clinical implementation. **Methods:** This prospective single-center study enrolled 382 participants: 142 with CRC, 62 with colorectal polyps, and 178 non-malignant controls. Plasma mSEPT9 was quantified by real-time PCR, urinary DiAcSpm by ELISA, and inflammatory indices from blood counts. Serum tumor markers (CEA, CA19-9, CA125, and AFP) were measured by immunoassay. Diagnostic accuracy was assessed using ROC analysis and multivariable logistic regression. **Results:** mSEPT9 (AUC 0.843) and DiAcSpm (AUC 0.831) demonstrated significantly higher diagnostic accuracy than CEA (AUC 0.660) and CA19-9 (AUC 0.649). A combined panel including mSEPT9, DiAcSpm, NLR, PLR, and LMR achieved an AUC of 0.947, with 85.9% sensitivity and 92.9% specificity. This panel also showed strong performance for early-stage CRC (AUC 0.905). **Conclusions:** A multimarker panel of biomarkers (mSEPT9, DiAcSpm, NLR, PLR, and LMR) provides a non-invasive diagnostic approach for CRC detection in a clinical case–control setting. Validation in asymptomatic screening populations is required before any screening claim can be made. These findings are associative; direct evidence that tumor hypoxia drives these biomarker changes was not obtained and requires future investigation.

## 1. Introduction

### 1.1. Global Colorectal Cancer Burden

Colorectal cancer (CRC) is a serious worldwide health problem. It is the third most common cancer and the second leading cause of cancer deaths. In 2020, there were more than 1.9 million new cases and about 935,000 deaths. By 2040, these numbers could increase by 50%, mainly due to aging populations and the wider adoption of Western lifestyles [1,2]. In high-income countries, CRC rates have dropped due to screening programs. In contrast, low- and middle-income countries (LMICs) are seeing a sharp rise in cases [3,4,5]. This is mostly because of limited screening; late diagnoses; and risk factors like obesity, lack of physical activity, and increased use of processed foods [6,7,8,9]. Research shows that LMICs are likely to experience the largest increase. By 2030, new CRC cases could rise by 60%, reaching about 2.2 million cases and 1.1 million deaths [10]. The main reason remains the lack of efficient screening methods that are both sensitive and affordable to address this crisis, in addition to aging populations, urbanization, and changing diets and lifestyles [11,12].

Colonoscopy remains the best way to diagnose CRC, but it needs many resources. It is invasive and not practical for large-scale use in resource-limited settings [13]. Stool-based tests are less invasive. However, too few people use them, and they are not sufficiently sensitive, especially in early-stage disease [14]. Therefore, there is a strong need for non-invasive, reliable biomarkers to assist in CRC diagnosis across different healthcare settings.

Tumor hypoxia is a critical aspect of colorectal cancer progression, reported to influence epigenetic regulation, metabolic adaptation, and immune responses primarily via hypoxia-inducible factor (HIF) signaling pathways [15,16]. Under hypoxic conditions, the oxygen-sensitive subunits HIF-1α and HIF-2α are stabilized and function as transcriptional regulators of genes involved in angiogenesis, metabolism, and cellular survival [16,17,18,19]. These signaling pathways are linked to changes in DNA methylation, metabolic reprogramming, and inflammatory signaling in colorectal cancer. Importantly, HIF-1β (also known as ARNT) is constitutively expressed and not regulated by hypoxia, but it serves as a dimerization partner required for HIF transcriptional activity [20,21].

### 1.2. Biological Framework for Biomarker Selection Based on the Prior Literature

In experimental models, tumor hypoxia has been reported to affect epigenetic regulation, metabolism, and immune responses in CRC [22]. When oxygen is low, HIF-1α and HIF-2α subunits become stable. They then regulate genes involved in blood vessel growth, metabolism, and cell survival [23]. These pathways are linked to changes in DNA methylation, metabolism, and inflammation in colorectal cancer [24,25]. HIF-1β, also called ARNT, is always present and acts as a required partner for HIF activity. It is not possible to directly measure tumor hypoxia, such as HIF-1α/2α levels or hypoxia gene signatures, in routine liquid biopsies. Therefore, our study does not directly measure hypoxia or HIF expression in patient tumors [26,27]. Instead, we examined whether a group of biomarkers, already linked to hypoxia-related processes, could work together in a non-invasive diagnostic test. These biomarkers include plasma methylated SEPT9 (mSEPT9, an epigenetic marker), urinary N^1^,N^12^-diacetylspermine (DiAcSpm, a metabolic marker), and systemic inflammatory indices (NLR, PLR, and LMR). We compared these to standard serum tumor markers. Previous studies have explored links between tumor metabolism (like FDG-PET uptake) and systemic inflammation in recurrent CRC [28]. In our study, the multimarker model is based on literature-reported links to hypoxia-related pathways

### 1.3. Study Rationale

Based on the convergent biology of signaling pathways previously linked to hypoxia in CRC, a streamlined multimarker panel integrating plasma mSEPT9, urinary DiAcSpm, and systemic inflammatory indices (NLR, PLR, and LMR) was prospectively designed. We also considered traditional serum markers (CEA, CA19-9, CA125, and AFP), which were evaluated as comparators but excluded from the final model due to limited diagnostic contribution and lack of mechanistic alignment with the literature-based hypoxia framework. This integrated strategy is intended to provide a biologically coherent approach to non-invasive CRC detection. However, the present study is diagnostic (case–control) and does not validate the panel for population screening. This panel is based on biomarkers with literature-based links to hypoxia-related pathways; however, this study does not directly measure tumor hypoxia or HIF signaling.

### 1.4. Biological Basis of Multimarker Panel (Literature-Informed)

This section outlines the biological processes that have been reported to connect hypoxia-related signaling to changes in epigenetics, metabolism, and the immune system in colorectal cancer [29,30]. Importantly, this study did not directly measure tumor hypoxia or HIF expression. The biomarkers included were selected based on earlier research linking them to pathways affected by hypoxic tumor environments [31]. Therefore, the multimarker model is intended to reflect biological processes that have been linked to hypoxic tumor microenvironments in prior studies, rather than to provide direct evidence of hypoxia in our patients.

### 1.5. mSEPT9—Epigenetic Changes and the Tumor Microenvironment (As Described in the Prior Literature)

The SEPT9 gene encodes a protein involved in cell division, cytoskeletal structure, and apoptosis. Aberrant methylation of the SEPT9 promoter is a well-recognized epigenetic hallmark of colorectal cancer. mSEPT9 is a useful biomarker because its methylation occurs at a highly specific site [32,33]; research shows that this abnormal methylation is restricted to a single CpG island in the SEPT9_v2 promoter. This precise location is diagnostically relevant, as a positive result typically originates from cancer cells rather than from stromal or benign inflammatory cells in the gut [34,35].

**Figure 1:**
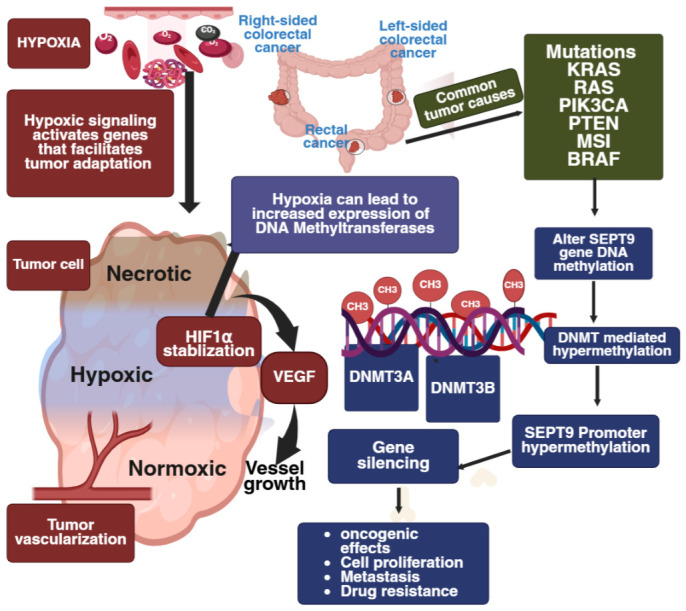
Literature-based model of epigenetic reprogramming linked to hypoxia. Bold black arrows indicate direct stimulation, activation, or progression toward downstream effects of hypoxia stabilization, and solid arrows represent established pathways to progression of CRC; Green text boxes indicate common CRC mutations (KRAS, RAS, PIK3CA, PTEN, MSI, BRAF). Blue text boxes represent progression to CRC from hypoxia and mutation. The light blue background in the tumor cell represents the hypoxic microenvironment. Tumor regions are color-coded: normoxic (light pink), hypoxic (light blue), and necrotic (grey). Tumor locations (right-sided colon, left-sided colon, rectal cancer) are shown in blue text.

Experimental studies have reported that this methylation pattern is influenced by the low-oxygen tumor microenvironment [36,37]. Hypoxia-inducible factors (HIFs), particularly HIF-1α and HIF-2α, regulate cellular responses to low oxygen levels [38,39]. In colorectal cancer models, HIFs have been shown to increase the activity of DNA methyltransferases (DNMTs), mainly DNMT1 and DNMT3A/3B [40]. These DNMTs may further enhance HIF activity by silencing negative regulators of hypoxic signaling, creating a potential feedback loop [41,42]. This HIF-DNMT interaction is thought to alter the cell’s epigenetic state, leading to targeted methylation and silencing of tumor suppressor genes, including SEPT9 [43,44]. The release of methylated SEPT9 DNA fragments into the bloodstream has been proposed as a detectable biomarker epigenetic change previously reported to be associated with hypoxia [32,45,46] (see Figure 1). Clinically, plasma mSEPT9 has been extensively validated as a non-invasive CRC marker, with the FDA-approved Epi proColon^®^ assay demonstrating superior sensitivity compared with conventional serum tumor markers, particularly in early-stage disease [47]. Previous studies have associated abnormal SEPT9 methylation with epigenetic changes related to hypoxia; however, the present study did not directly evaluate HIF signaling. Consequently, mSEPT9 should be regarded as a circulating epigenetic biomarker that may reflect tumor-associated molecular alterations, including those reported to be linked to hypoxia [48].

This schematic summarizes published mechanisms linking tumor hypoxia to DNA methylation changes. Colorectal tumors contain regions of normal oxygenation, hypoxia, and necrosis due to inadequate blood supply. Under hypoxic conditions, HIF-1α is stabilized and translocates to the nucleus, where it upregulates target genes including VEGF (promoting abnormal angiogenesis) and DNA methyltransferases (DNMT1, DNMT3A, and 3B). Increased DNMT activity leads to hypermethylation of tumor suppressor gene promoters, such as SEPT9. Methylated SEPT9 DNA fragments are released into the circulation, enabling non-invasive detection. These epigenetic changes can co-occur with common CRC mutations (e.g., KRAS, BRAF, PIK3CA, and MSI).

Note: This figure is based on the prior literature and is provided to illustrate the biological rationale for biomarker selection. No direct measurement of HIF-1α, HIF-2α, or tissue oxygen levels was performed in the patient samples in this study.

### 1.6. The Role of the Tumor Microenvironment (As Described in the Prior Hypoxia Literature) in Enhancing DiAcSpm-Associated Metabolic Reprogramming

Colorectal cancer (CRC) tumors often develop hypoxic regions, arising when rapid cellular proliferation outpaces neovascularization. Experimental studies have shown that hypoxia-inducible factor 1α (HIF-1α) mediates changes in gene expression during polyamine synthesis and metabolism, enabling cellular adaptation and destabilizing non-coding RNAs, thus mediating the Warburg effect in CRC carcinogenesis progression [49,50]. Under hypoxic conditions, cellular metabolism shifts toward pathways supporting polyamine biosynthesis, increasing the availability of substrates required for SSAT-mediated acetylation and subsequent DiAcSpm formation. Notably, a study using CRC cell lines cultured under hypoxia reported that these metabolic alterations may serve as potential biomarkers [51].

**Figure 2:**
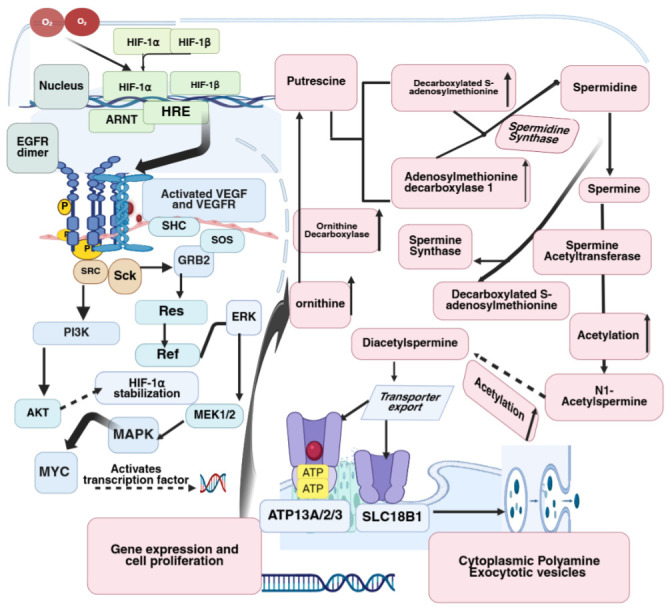
Literature-based model of signaling in polyamine metabolism influenced by hypoxia in CRC. Black arrows indicate stimulation, activation, or progression toward downstream consequences. Dashed arrows indicate direct transition to effects or downstream consequences. Green box text denotes upregulated hypoxia; blue box text denotes downstream activation of MYC transcription regulated factors; Red box text denotes upregulation of Diacetylspermine production.

Polyamines—including putrescine, spermidine, and spermine—are critical regulators of cellular proliferation and differentiation. In CRC, polyamine metabolism is significantly dysregulated, a phenomenon linked to hypoxia-associated signaling pathways [52,53]. Under hypoxic conditions, HIF-2α activation upregulates c-MYC transcription [50,54,55], which in turn induces key enzymes such as ornithine decarboxylase (ODC) and spermidine/spermine N^1^-acetyltransferase (SAT1) [56]. This metabolic reprogramming also affects immunometabolism and immune regulation, increasing spermine production and acetylation, leading to the formation of N^1^,N^12^-diacetylspermine [57] (DiAcSpm) (see Figure 2).

DiAcSpm is a chemically stable metabolite that is readily excreted in urine. This provides a non-invasive indicator of metabolic dysregulation. Urinary DiAcSpm can be quantified by ELISA or LC–MS/MS. It has shown consistent diagnostic performance in CRC, with reported sensitivities exceeding 70% and specificities above 80%. DiAcSpm often outperforms classical serum tumor markers [58]. These properties make DiAcSpm a robust metabolic biomarker linked to tumor adaptation processes reported in the literature and associated with hypoxia. Researchers at Karolinska University Hospital found DiAcSpm levels were 8.1 times higher in CRC patient tissues, underscoring the marker’s significance [59]. It is important to note that, as in the mSEPT9 section, this study did not directly assess HIF-1α, HIF-2α, or tissue oxygen in patient samples. This biological model is based on prior published experimental evidence and is intended to provide a rationale for biomarker selection. Urinary DiAcSpm should therefore be interpreted as a metabolite whose elevation is associated with CRC status. It is not a direct indicator of tumor hypoxia.

This schematic shows how hypoxia signaling connects to polyamine metabolism and the production of N^1^,N^12^-diacetylspermine (DiAcSpm). In low-oxygen conditions, HIF-1α becomes stable and moves into the nucleus. Inside the nucleus, HIF-1α joins with HIF-1β (ARNT). This complex binds to hypoxia response elements (HREs) and activates genes such as VEGF. These genes support the growth of new blood vessels and trigger oncogenic pathways, such as EGFR/PI3K/AKT and MAPK/ERK. These pathways further increase HIF activity and MYC-dependent gene expression.

These integrated signals drive metabolic reprogramming of polyamine biosynthesis. Ornithine is converted to putrescine by ornithine decarboxylase (ODC) and then sequentially to spermidine and spermine via spermidine synthase and spermine synthase, using decarboxylated S-adenosylmethionine (dcSAM) generated by AMD1. Spermine acetyltransferase (SAT1) catalyzes acetylation, producing N^1^-acetylspermine and ultimately N^1^,N^12^-diacetylspermine (DiAcSpm). Acetylated polyamines are exported via transporters (e.g., SLC18B1 and ATP13A2/3) or exocytic vesicles and become detectable in blood and urine.

Note: This figure is based on the prior literature and is provided to illustrate the biological rationale for selecting DiAcSpm as a metabolic biomarker. No direct measurement of HIF-1α, HIF-2α, or tissue oxygen levels was performed in the patient samples included in this study. Urinary DiAcSpm levels are reported as statistical associations with CRC status, not as direct evidence of tumor hypoxia.

### 1.7. Neutrophil-to-Lymphocyte Ratio (NLR), Platelet-to-Lymphocyte Ratio (PLR), and Lymphocyte-to-Monocyte Ratio (LMR) as Described in the Prior Literature on the Tumor Microenvironment

Experimental studies indicate that hypoxia within the tumor microenvironment promotes the recruitment and functional reprogramming of innate immune cells, particularly macrophages and neutrophils [60]. Hypoxia-associated chemokines and growth factors, such as CCL2, CXCL12, VEGF, and macrophage colony-stimulating factor, drive monocyte infiltration and polarization toward tumor-associated macrophages [60,61]. These macrophages contribute to angiogenesis, extracellular matrix remodeling, and immunosuppression by secreting cytokines, matrix metalloproteinases, and pro-angiogenic mediators. This process reinforces tumor progression and shapes systemic inflammatory responses that are detectable in peripheral blood indices.

Elevated NLR and PLR result from cytokines in the tumor microenvironment that stimulate granulopoiesis and thrombopoiesis while suppressing lymphocyte function [62,63,64]. The platelet-to-lymphocyte ratio correlates positively with circulating tumor cell counts [65], and both NLR and PLR are significantly associated with poor tumor differentiation [66]. The LMR serves as an indicator of immune competence and reflects tumor-associated macrophage activity, as monocytes are recruited to the tumor and lymphocyte counts decrease due to cytokine-mediated immunosuppression. A low LMR independently predicts worse progression-free and overall survival [67].

Systemic inflammatory indices derived from routine blood counts, including NLR, PLR, and LMR, capture these immune alterations previously reported to be associated with hypoxia, which are characterized by increased NLR and PLR, and reduced LMR [60,68,69]. Although these indices are well established as prognostic markers in CRC, their diagnostic utility within an integrated hypoxia-associated biomarker framework remains insufficiently explored.

Note: As with the epigenetic and metabolic biomarkers, this study did not directly measure tumor hypoxia, HIF expression, or tissue oxygen levels. The association between systemic inflammatory indices and hypoxia is based on the prior literature; in this study, these indices are interpreted as statistical correlates of CRC status rather than as direct markers of tumor hypoxia.

### 1.8. Classical Serum Tumor Markers

Conventional serum tumor antigens, such as carcinoembryonic antigen (CEA), carbohydrate antigen 19-9 (CA19-9), carbohydrate antigen 125 (CA125), and alpha-fetoprotein (AFP), are widely used as clinical comparators. However, these markers demonstrate limited sensitivity for early-stage CRC, often below 40%, and lack direct mechanistic links to tumor biology associated with hypoxia in the literature [70]. Consequently, their diagnostic performance is insufficient for use as standalone tools for early detection. In this study, these markers were evaluated as comparators but were excluded from the final multimarker model due to limited diagnostic contribution and lack of alignment with the hypoxia-associated framework.

## 2. Materials and Methods

Below, we present a detailed participant flow diagram following STARD guidelines.


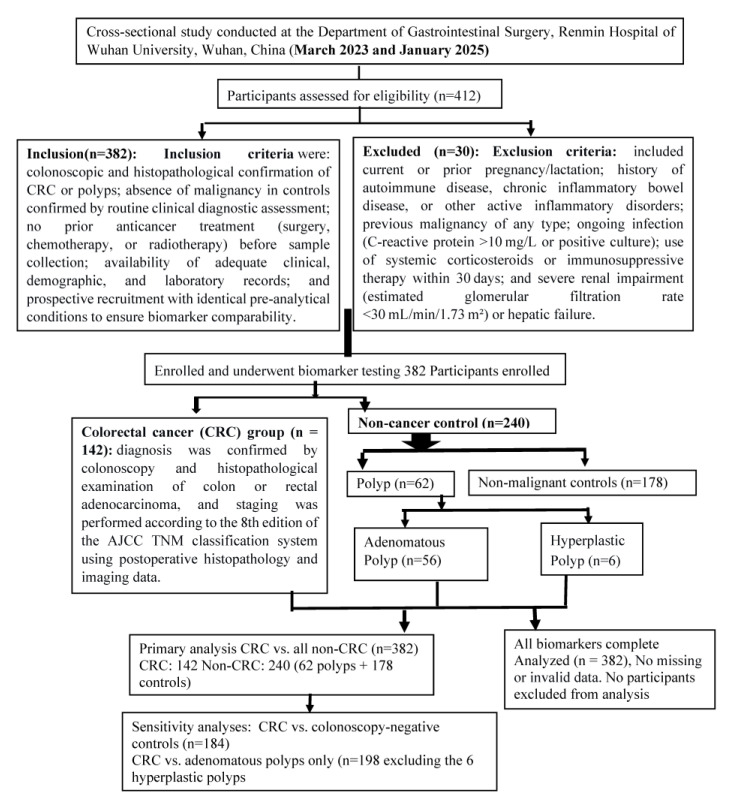


**Non-Invasive multi-analyte flowchart for CRC**

Patient (CRC)

│

├─▶ **Whole Blood (EDTA) **

│ ├─▶ Centrifugation → Plasma (~55%) / Buffy Coat (~1%) / RBC Pelleted (~45%)

│ │ ├─▶ Plasma → cfDNA Extraction → Methylation-Specific QM-PCR (*SEPT9*)

│ │ └─▶ Buffy Coat → Flow Cytometry → NLR/PLR/LMR Calculation

│ └─▶ Serum → Classical Markers (CEA, CA19-9, CA125, AFP) via immunoassays (CLIA)

│

└─▶ **First-Morning Urine**

└─▶ DiAcSpm Extraction → ELISA (Quantitative Analysis)

### 2.1. Study Design and Participants

Between March 2023 and January 2025, a total of 382 participants were prospectively enrolled at the Department of Gastrointestinal Surgery, Renmin Hospital of Wuhan University, under an institutional ethics committee-approved protocol (approval No. WDRY2025-K158). Written informed consent was obtained from all participants before inclusion.

The study cohort comprised 142 patients with histologically confirmed colorectal cancer (CRC), 62 patients with colorectal polyps, and 178 non-malignant controls consisting of individuals treated for benign conditions such as hernia or hemorrhoids without evidence of malignancy.

All CRC diagnoses were established through colonoscopic evaluation followed by histopathological confirmation. The colorectal polyp group (*n* = 62) included histologically confirmed adenomatous polyps (tubular, tubulovillous, and villous), serrated lesions, and hyperplastic polyps. Hyperplastic polyps are generally non-neoplastic but were included to reflect the full spectrum of colorectal lesions encountered in clinical practice. A sensitivity analysis excluding all polyps from the non-CRC comparator group (including hyperplastic polyps) yielded similar diagnostic performance (AUC 0.971 vs. primary 0.947), indicating that inclusion of hyperplastic polyps did not materially bias the results.

Control participants were recruited from patients undergoing clinical evaluation for non-malignant surgical conditions. The absence of colorectal malignancy was determined through routine clinical assessment, including colonoscopy when clinically indicated, imaging studies, laboratory investigations, and specialist evaluation. No control participant demonstrated clinical, endoscopic, or pathological evidence of CRC. Among the 178 non-cancer controls, 42 (23.6%) underwent colonoscopy as part of their clinical evaluation; the remaining controls were deemed free of colorectal neoplasia based on clinical examination, imaging, and follow-up (minimum 12 months) where available, reflecting a real-world clinical diagnostic setting rather than a population-based screening cohort in this study.

This cross-sectional study was conducted in accordance with the Declaration of Helsinki (2013 revision) and followed STARD 2015 [71] and TRIPOD reporting guidelines for diagnostic biomarker studies. Recruitment procedures, biospecimen collection, and sample handling adhered to institutional ethical and quality standards. Tumor staging was performed using the tumor–node–metastasis (TNM) classification according to the American Joint Committee on Cancer (AJCC, 8th edition) and lymph node ratio (LNR) based on a cutoff of ≥0.20 according to established prognostic thresholds in colorectal cancer (Berger et al., J Clin Oncol 2005; Park et al., Ann Coloproctol 2013) [72,73]. All biological samples were obtained before initiation of any therapeutic intervention.

### 2.2. Sample Processing and Storage

Fasting peripheral blood samples (10 mL) were collected into K_2_-EDTA tubes and centrifuged at 1350× *g* for 10 min within 2 h of collection. Plasma was aliquoted and stored at −80 °C until circulating cell-free DNA (cfDNA) analysis. First-morning urine samples were processed within 4 h of collection, aliquoted, and stored at −80 °C, and samples underwent no more than two freeze–thaw cycles to preserve analyte stability.

Urinary N^1^, N^12^-diacetylspermine (DiAcSpm) concentrations were reported as absolute values (ng/mL) without creatinine normalization to avoid confounding related to renal function, hydration status, or systemic inflammation.

### 2.3. Detection and Quantification of Plasma Methylated SEPT9

Plasma cfDNA was extracted from ≥4 mL EDTA plasma using a magnetic bead-based kit (Namagene, Wuhan, China) optimized for low-abundance methylated DNA recovery. DNA quantity and fragment distribution were verified before bisulfite conversion. Bisulfite treatment (Biyuntian, Shanghai, China) selectively converted unmethylated cytosines to uracil while preserving methylated cytosines, enabling methylation-specific amplification. Converted DNA (20 μL) was obtained after thermal cycling (95 °C for 3 min; 12 cycles of 95 °C for 30 s and 70 °C for 10 min) and purification.

Methylated SEPT9 (mSEPT9) was quantified by quantitative methylation-specific PCR (qMSP) using a Bori real-time PCR system (Bori, Beijing, China). Reactions (20 μL) contained 1 μL bisulfite-converted DNA and dual-labeled probes detecting methylated (FAM) and unmethylated (HEX) alleles, with ACTB as an internal control. Cycling conditions were 95 °C for 10 min, followed by 40 cycles of 95 °C for 15 s, 53 °C for 30 s, and 60 °C for 30 s.

Methylation scores were calculated as 100/[1 + 2^ΔCt^], where ΔCt (Ct_m_ − Ct_u_) reflects the relative abundance of methylated (Ct_m_) versus unmethylated (Ct_u_) DNA. Lower Ct_m_ values indicate higher methylated cfDNA levels. Quantitative accuracy was verified using calibration curves derived from standards with known methylation fractions [74,75]. Assay performance was validated using calibration curves derived from known methylation standards.

### 2.4. Quantification of Urinary DiAcSpm by Competitive ELISA

Urinary DiAcSpm concentrations were measured using a competitive enzyme-linked immunosorbent assay (ELISA) kit (Shanghai Lianzu Biotechnology, Shanghai, China), following the manufacturer’s protocol. Each 96-well plate included six standard concentrations (0–40 ng/mL), prepared in duplicate, along with internal quality controls.

Urine samples stored at −80 °C were diluted 1:5 in assay buffer to align with the linear detection range (1–50 ng/mL; limit of detection 0.1 ng/mL). Standard curves were generated using four-parameter logistic regression. In brief, DiAcSpm in samples competed with horseradish peroxidase (HRP)-conjugated DiAcSpm for binding to immobilized monoclonal antibodies. After incubation and washing, tetramethylbenzidine (TMB) substrate was added, and the reaction was stopped to generate a colorimetric signal.

Optical density was measured at 450 nm and was inversely proportional to DiAcSpm concentration. All incubations were performed at 37 °C in the dark to maintain assay consistency. Intra- and inter-assay coefficients of variation were ≤8% and ≤10%, respectively. Samples exceeding the assay range were diluted and re-analyzed. DiAcSpm values were reported as absolute concentrations (ng/mL) without creatinine normalization to avoid confounding from physiological or pathological factors [76,77]. However, we acknowledge that urine dilution can affect absolute concentrations. Because we did not measure urinary creatinine, we cannot normalize the values. This is a limitation that may influence the interpretation of absolute DiAcSpm levels (see Section 4).

Detailed assay procedures and calibration plots for mSEPT9 and DiAcSpm are provided in the Supplementary Methods (Files S1 and S2, respectively).

### 2.5. Peripheral Inflammatory Blood Indices

Fasting venous blood samples (3–5 mL) were collected between 07:00 and 09:00 to minimize circadian variation. Complete blood counts were analyzed using Sysmex XN-Series hematology analyzers (XN9000; Sysmex Corporation, Kobe, Japan) based on fluorescence flow cytometry.

Neutrophil-to-lymphocyte ratio (NLR), platelet-to-lymphocyte ratio (PLR), and lymphocyte-to-monocyte ratio (LMR) were calculated from absolute cell counts. These indices were evaluated as systemic indicators of hypoxia-associated immune activation.

### 2.6. Serum Tumor Marker Measurement

Serum was obtained from 3–5 mL venous blood after clotting at room temperature for 30 min and centrifugation at 3000 rpm for 5 min. Serum carcinoembryonic antigen (CEA), carbohydrate antigen 19-9 (CA19-9), carbohydrate antigen 125 (CA125), and alpha-fetoprotein (AFP) were quantified using Siemens ADVIA Centaur^®^ XP/XPT immunoassay systems (Siemens Healthineers, Erlangen, Germany), according to the manufacturer’s instructions.

Thresholds for abnormal values were defined as follows: CEA ≥ 5 ng/mL, CA19-9 ≥ 30 U/mL, CA125 ≥ 24 U/mL, and AFP ≥ 7 ng/mL. These markers were evaluated as comparators but were excluded from the final multimarker model due to limited diagnostic contribution and lack of mechanistic association with hypoxia signaling.

### 2.7. Determination of Diagnostic Cutoff Values

Optimal cutoff values for biomarkers were determined using ROC curve analysis. The selected thresholds were ≥10.01% for plasma mSEPT9 and ≥32.32 ng/mL for urinary DiAcSpm, maximizing sensitivity and specificity for CRC detection. These cutoffs were derived from the same dataset without an independent training/validation split; therefore, the reported performance metrics may be optimistic and require external validation (see Section 4). Raw data for mSEPT9 methylation scores, urinary DiAcSpm concentrations, and inflammatory indices (NLR, PLR, LMR) are provided in Data S1, Data S2, and Data S3, respectively.

### 2.8. Statistical Analysis

Statistical analyses were conducted using IBM SPSS Statistics (version 26.0, IBM Corp., Armonk, NY, USA) and R (version 4.2.1, R Foundation for Statistical Computing, Vienna, Austria), with the pROC package. The Kolmogorov–Smirnov test was used to assess the distribution of the data.

Continuous variables are reported as medians with interquartile ranges (IQRs). Comparisons between two groups were performed using the Mann–Whitney U test, while the Kruskal–Wallis test was applied for three or more groups, with Dunn–Bonferroni post hoc correction when appropriate. Categorical variables were presented as counts and percentages and compared using the Chi-square test or Fisher’s exact test, as appropriate. Paired comparisons of diagnostic performance between biomarkers were conducted using McNemar’s test.

Diagnostic accuracy was assessed using receiver operating characteristic (ROC) curve analysis. The area under the curve (AUC) was calculated with 95% confidence intervals, and comparisons between ROC curves were conducted using DeLong’s test. Sensitivity, specificity, positive predictive value (PPV), and negative predictive value (NPV) were calculated with corresponding 95% confidence intervals.

For all primary ROC analyses comparing CRC versus non-CRC, the non-CRC group included both colorectal polyp patients (*n* = 62) and non-malignant controls (hernia and hemorrhoid patients, *n* = 178), unless otherwise specified. Subgroup-specific ROC analyses (e.g., early vs. advanced stage, or the sensitivity analysis restricted to colonoscopy-negative controls) are indicated where presented.

All statistical tests were two-sided, and *p*-values less than 0.05 were considered statistically significant. Model robustness and overfitting were addressed through internal validation using bootstrap resampling (1000 iterations). Optimism-corrected performance estimates were reported to provide more conservative and reliable measures of diagnostic accuracy.

The D4 panel (mSEPT9, DiAcSpm, NLR, PLR, and LMR) was pre-specified based on published biological links to hypoxia-associated pathways, not selected by data-driven comparison among all possible marker combinations. The bootstrap internal validation (1000 iterations) was applied only to the final D4 model after all model and cutoff decisions had been made; therefore, the optimism correction does not account for model-selection or cutoff-selection bias. The absence of an external validation cohort is acknowledged as a major limitation.

## 3. Experimental Results and Analysis

A total of 382 participants were prospectively enrolled between March 2023 and January 2025 and included in the final analysis, comprising 142 patients with histologically confirmed colorectal cancer (CRC), 62 patients with colorectal adenomatous polyps, and 178 non-cancer controls diagnosed with inguinal hernia or hemorrhoids. The overall cohort included 203 males and 179 females, with a mean age of 58 years, reflecting a clinically representative population encountered in gastrointestinal surgical practice. Detailed demographic distribution according to diagnostic category, sex, and age group is presented in Table 1, while clinicopathological and lifestyle characteristics are summarized in Table 2.

Age distribution differed across diagnostic groups, with CRC patients presenting at an older mean age (65 years) compared with non-cancer controls, consistent with the established age-related risk profile of colorectal carcinogenesis. Sex distribution was balanced across groups and showed no statistically significant difference (*p* = 0.992), indicating minimal sex-related selection bias within the study population. Age stratification demonstrated a progressive increase in CRC frequency in older age categories, particularly among individuals aged 60–69 and ≥70 years, whereas younger age groups were predominantly represented in control subgroups, especially hemorrhoid patients.

Tumor localization among CRC cases showed heterogeneous anatomical distribution, with lesions identified in the right colon (*n* = 38), left colon (*n* = 65), and rectum (*n* = 39), supporting the inclusion of tumors arising from distinct embryologic and molecular colorectal segments. Disease stages spanned the full clinical spectrum (stages I–IV), with 13.4% of patients presenting with metastatic disease at diagnosis, reflecting real-world clinical presentation rather than screening-detected cohorts.

Pathological evaluation revealed that ulcerative morphology represented the predominant gross tumor type (56.3%), followed by polypoid lesions. Depth of invasion indicated advanced local disease in most cases, with more than 80% of tumors extending beyond the muscularis propria into pericolic tissues, serosa, or adjacent structures. Indicators of aggressive tumor biology were frequently observed, including lymphovascular invasion in approximately half of cases, perineural invasion in 41.5%, and lymph node involvement in 45.1% of patients. Tumor budding analysis demonstrated a distribution across low-, intermediate-, and high-grade categories, supporting biological heterogeneity within the CRC cohort. Molecular characterization showed a predominance of mutant-pattern p53 expression, while the median Ki-67 proliferation index of 60% (IQR 50–70) indicated high proliferative activity consistent with malignant epithelial tumors.

The colorectal polyp group (*n* = 62) included histologically confirmed adenomatous polyps (tubular, tubulovillous, and villous), serrated lesions, and hyperplastic polyps. Hyperplastic polyps are generally non-neoplastic but were included to reflect real-world clinical lesion distribution. Inclusion of this intermediate group (including adenomas with varying dysplasia grades) enabled evaluation of biomarker behavior during early neoplastic transformation preceding invasive cancer development.

Lifestyle characteristics demonstrated selective differences between groups. Alcohol consumption was significantly more common among CRC patients compared with other diagnostic categories (*p* = 0.007), suggesting a potential association with disease risk within this cohort. In contrast, smoking status and family history of cancer did not differ significantly across groups (*p* = 0.265 and *p* = 0.811, respectively), indicating that these factors were unlikely to confound biomarker comparisons in subsequent analyses.

Among the colorectal polyp cohort (*n* = 62), the majority were adenomatous lesions, including tubular, tubulovillous, and villous subtypes, with a subset demonstrating high-grade dysplasia. Detailed histological distribution is presented in Table 2.

Overall, the demographic balance, broad stage representation, and detailed pathological characterization confirm that the study population captures the clinical and biological diversity of colorectal disease progression, ranging from premalignant adenomas to invasive carcinoma and benign surgical conditions. This structured cohort provides an appropriate foundation for evaluating non-invasive biomarker performance across the continuum of colorectal tumorigenesis.

### 3.1. Diagnostic Yield Across Clinical Subgroups Between mSEPT9 and DiAcSpm Biomarkers

**Figure 3:**
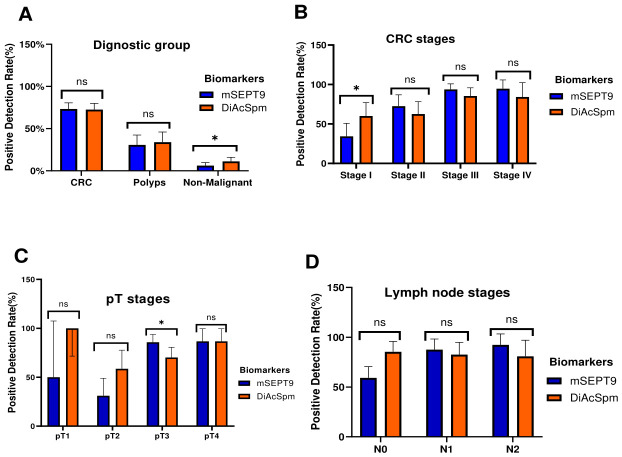
Positive detection rates of mSEPT9 and DiAcSpm across clinical subgroups. Positive detection rates of mSEPT9 and DiAcSpm across clinical subgroups with statistical comparisons. (**A**) Diagnostic categories (CRC, polyps, and controls); (**B**) tumor stages; (**C**) pT stage; and (**D**) nodal status. Bars represent the proportion of biomarker-positive cases with 95% Wilson confidence intervals. Asterisks indicate statistical significance between mSEPT9 and DiAcSpm within each diagnostic group (McNemar’s test *p*-values). Significance thresholds are indicated as ns, not significant; * *p* < 0.05.

Both plasma methylated SEPT9 and urinary N^1^, N^12^-diacetylspermine (DiAcSpm) showed strong and complementary results for diagnosing colorectal cancer (CRC). The detection rates were close: mSEPT9 detected 73.2% of CRC cases, while DiAcSpm detected 72.5%, both well above chance (Figure 3A). There were a few false positives among patients with colorectal polyps and almost none in the non-malignant controls, showing that the tests are highly specific. Table 3 shows there is a significant difference in positive detection rates between the two biomarkers (*p* < 0.05).

Across different cancer stages, both biomarkers were more likely to be positive as the tumor progressed. mSEPT9 positivity increased from 34.3% in stage I to 94.7% in stage IV, suggesting greater epigenetic changes. Urinary DiAcSpm also increased, from 60.0% in stage I to 84.2% in stage IV (Figure 3B), reflecting polyamine activity previously linked to hypoxia-related pathways. Looking at how deeply the tumor invaded (Figure 3C), mSEPT9 positivity rose from 31.0% in pT2 to 86.7% in pT4, and DiAcSpm rose from 58.6% to 86.7%. All six pT1 tumors tested positive for DiAcSpm, suggesting it may be sensitive to early metabolic changes, though the sample size is small. Nodal status (Figure 3D) showed that both biomarkers had nearly identical detection rates across all stages, indicating they are similarly effective. Using both markers together improves overall diagnostic accuracy.

### 3.2. Comparative Analysis of mSEPT9 and DiAcSpm Biomarker Positivity and Negativity Status: Clinicopathological Correlations

Clinicopathological analyses revealed biological differences between these two investigated biomarkers. Table S1 gives a summary; mSEPT9 positivity was strongly linked to signs of more aggressive tumors. It was found more often in patients aged 60 or older (*p* < 0.001) and in larger tumors (*p* < 0.001). mSEPT9 positivity also increased with higher TNM stage, deeper tumor invasion, lymph node involvement, and distant metastasis, showing a clear link to disease progression. There were also connections with poorer tumor differentiation (*p* = 0.042) and lymph node invasion (*p* < 0.001). This suggests that mSEPT9 reflects epigenetic changes tied to invasive and metastatic potential. On the other hand, there were no significant links with tumor location, overall appearance, vascular invasion, or perineural invasion. This means that methylation status primarily reflects tumor aggressiveness, not its location.

In comparison, the clinicopathological links for urinary DiAcSpm (Table S2) point to a more local biological role. DiAcSpm positivity was associated with higher tumor stage (*p* = 0.018) and deeper invasion (pT stage, *p* = 0.040), suggesting it is sensitive to tumor growth at the primary site. Unlike mSEPT9, DiAcSpm was not significantly associated with lymph node or distant metastasis, tumor differentiation, vascular invasion, or perineural invasion. Higher positivity was also observed in patients aged 60 or older (*p* < 0.001), but sex and tumor location did not differ significantly.

However, the convergence of these independent, biologically linked biomarkers, mSEPT9 and DiAcSpm, explains their similar diagnostic performance. It also highlights their distinct clinicopathological associations. This approach enables non-invasive detection and simultaneously provides biological stratification within a unified literature-informed diagnostic framework.

### 3.3. McNemar’s Test Comparison for Significance Between (mSEPT9 and DiAcSpm) Biomarkers in Different Clinical Subgroups

McNemar’s analyses across clinical subgroups in Table 3 below confirmed largely concordant diagnostic behavior between the biomarkers (mSEPT9 and DiAcSpm). No significant discordance was observed across most tumor stages or nodal categories, whereas significant differences emerged within the non-malignant control group (*p* = 0.031) and at the pT3 invasion stage (*p* = 0.0197), and tumor stage I (*p* = 0.050 *), indicating almost no variation in detection behavior of these two biomarkers except in a few cases of disease status and tumor progression. These comparisons evaluated paired positive–negative classifications within the same individuals. All staging classifications were defined according to the American Joint Committee on Cancer (AJCC) 8th edition TNM system. Significance threshold: *p* < 0.05.

### 3.4. Comparison of mSEPT9 and DiAcSpm Biomarker Positivity and Negativity Status Correlating with Inflammatory Indices

To evaluate whether tumor-derived biomarkers are associated with systemic inflammatory status, relationships between mSEPT9, DiAcSpm, and circulating inflammatory indices including neutrophil-to-lymphocyte ratio (NLR), platelet-to-lymphocyte ratio (PLR), and lymphocyte-to-monocyte ratio (LMR), were analyzed in colorectal cancer patients (Table 4).

Patients were stratified independently according to biomarker positivity, generating two separate binary comparisons: mSEPT9-positive vs. mSEPT9-negative and DiAcSpm-positive vs. DiAcSpm-negative.

Because the inflammatory indices were non-normally distributed, comparisons between the positive and negative groups for each biomarker were performed using the Mann–Whitney U test (two independent groups). Thus, each reported *p* value represents a pairwise comparison within a single biomarker classification, rather than a simultaneous comparison across four groups.

mSEPT9-positive patients exhibited significantly higher NLR (median 3.05 [IQR 2.23–3.86]) and PLR (181.25 [137.79–228.96]) compared with mSEPT9-negative patients (NLR 2.20 [1.78–2.23]; PLR 126.19 [107.38–166.02]; both *p* < 0.001). Conversely, LMR was significantly lower among mSEPT9-positive individuals (2.49 [1.92–3.28] vs. 3.50 [2.66–4.89]; *p* < 0.001). A comparable pattern was observed for DiAcSpm stratification. DiAcSpm-positive patients demonstrated elevated NLR (2.83 [2.12–3.69]) and PLR (174.26 [123.51–232.65]) together with reduced LMR (2.65 [1.93–3.42]) relative to DiAcSpm-negative patients (NLR 2.24 [1.81–2.96]; PLR 128.19 [108.06–168.49]; LMR 3.43 [2.60–4.88]; all *p* < 0.001).

These findings indicate that biomarker positivity is consistently associated with a systemic inflammatory profile characterized by increased neutrophil- and platelet-dominant responses alongside reduced lymphocyte-associated immune balance. While hypoxia-related mechanisms have been proposed in prior experimental studies, showing that tumor progression is accompanied by proinflammatory cytokines including IL-6, IL-8, TNF-α, and VEGF production and myeloid cell expansion, processes that elevate NLR and PLR while reducing lymphocyte-dependent immune activity [51,52,53], the present analysis demonstrates statistical associations between circulating tumor biomarkers and host inflammatory remodeling. Collectively, the results support integration of molecular (mSEPT9), metabolic (DiAcSpm), and inflammatory indices within a combined diagnostic framework for colorectal cancer.

### 3.5. Comparative Distributions of mSEPT9, DiAcSpm, Inflammatory, and Classical Biomarkers Across Diagnostic Categories

**Figure 4:**
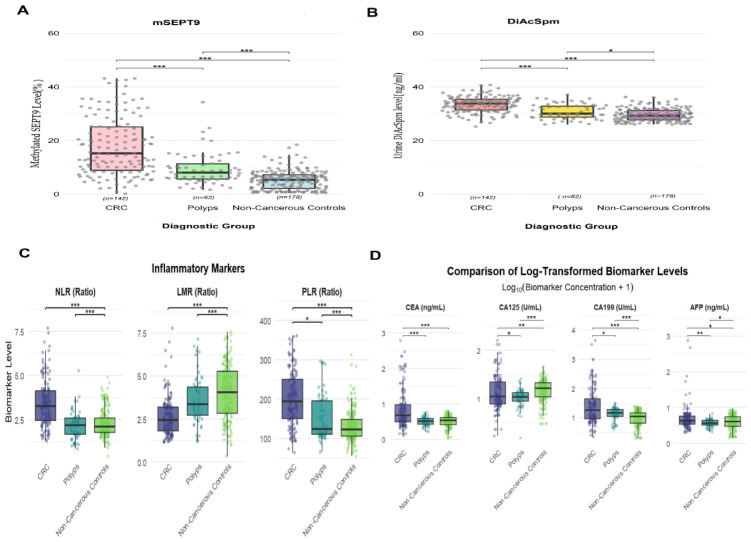
Comparative distributions of epigenetic, metabolic, inflammatory, and classical biomarkers across diagnostic categories. Comparative distributions of epigenetic, metabolic, inflammatory, and classical biomarkers across diagnostic categories. (**A**) mSEPT9; (**B**) DiAcSpm; (**C**) NLR, PLR, and LMR; and (**D**) log-transformed CEA, CA125, CA19-9 and AFP. Data are shown as boxplots with jittered individual points; horizontal bars indicate medians. Log transformation improved normality for classical markers. Black asterisks indicate between-group comparison significance, assessed using Kruskal–Wallis with Dunn’s post hoc test and Benjamini–Hochberg correction. Significance thresholds are indicated as * *p* < 0.05; ** *p* < 0.01; *** *p* < 0.001.

Non-parametric analyses found clear differences in biomarker levels among colorectal cancer (CRC), colorectal polyp, and non-cancer control groups (Table S3; Figure 4A). Plasma mSEPT9 methylation showed the most distinct separation. CRC patients had much higher methylation percentages (median = 15.2%, IQR 8.89–25.07) than those with polyps (8.05%, IQR 5.62–11.31) or controls (5.35%, IQR 2.15–7.25; *p* < 0.001). These results suggest a gradual epigenetic transition from adenoma to carcinoma and support mSEPT9 as a tumor marker, consistent with earlier reports of DNA methylation changes in colorectal cancer.

Urinary N^1^, N^12^-diacetylspermine (DiAcSpm) levels (Figure 4B) also showed a similar pattern: CRC patients had higher levels (33.81 ng/mL, IQR 31.42–35.37) than those with polyps (30.03 ng/mL, IQR 28.73–32.84) or controls (29.28 ng/mL, IQR 27.71–31.21; *p* < 0.001). While the difference was smaller than for mSEPT9, this gradual increase suggests changes in polyamine metabolism as cancer develops, consistent with earlier findings linking higher polyamine turnover to tumor growth and metabolic changes.

Systemic inflammatory markers (Figure 4C) also varied between groups. CRC patients had higher neutrophil-to-lymphocyte ratios (NLRs) and platelet-to-lymphocyte ratios (PLRs) but lower lymphocyte-to-monocyte ratios (LMRs) (all *p* < 0.001). These results suggest a more proinflammatory, imbalanced immune state in CRC. These blood changes likely show the body’s inflammatory response to tumor growth, rather than directly measuring hypoxia.

Classical serum tumor markers (CEA, CA19-9, CA125, and AFP; Figure 4D) did not clearly separate the groups, even though the differences were statistically significant (*p* < 0.001). This means they are less specific for early tumor-related changes. Increases in these markers are likely due to tumor size, tissue breakdown, or mucin production, rather than specific molecular changes.

These findings show that CRC is characterized by changes in epigenetic, metabolic, and inflammatory markers. While previous studies [78] connect these changes to hypoxia in tumors, this study only looks at the measurable biomarker patterns, not direct hypoxia levels. The combined biomarker profile offers a practical, integrated approach to better distinguish between cancer, precancer, and non-cancer cases than using traditional antigen markers alone.

### 3.6. Comparison of Classical Tumor Markers and Inflammatory Indices According to Clinico-Pathological Characteristics

Comparative analyses were performed to evaluate the relationships between classical tumor markers, systemic inflammatory indices, and clinico-pathological characteristics among colorectal cancer (CRC) patients (Table S4). Overall, systemic inflammatory indices demonstrated stronger and more consistent associations with indicators of tumor progression than conventional serum tumor markers.

The neutrophil-to-lymphocyte ratio (NLR) showed a clear association with disease severity. NLR increased progressively with advancing TNM stage (*p* < 0.001) and was significantly associated with greater depth of tumor invasion (T stage; *p* = 0.015), nodal involvement (*p* = 0.001), and the presence of distant metastasis (*p* = 0.007). These findings indicate that elevated NLR reflects increasing systemic inflammatory activation accompanying tumor progression. Similarly, the platelet-to-lymphocyte ratio (PLR) was significantly higher in patients with lymph node metastasis (*p* = 0.002) and poorer histological differentiation (*p* = 0.009), suggesting an association between platelet-mediated inflammatory responses and aggressive tumor biology. In contrast, the lymphocyte-to-monocyte ratio (LMR) decreased significantly in patients with nodal metastasis (*p* = 0.033) and in older patients (*p* < 0.001), consistent with reduced immune surveillance capacity during disease advancement.

Importantly, these inflammatory indices represent systemic host responses measurable through routine hematological testing. Although prior experimental studies have linked inflammatory remodeling to hypoxia-related signaling pathways in tumors, the present study did not directly measure hypoxia or hypoxia-inducible factor expression. Therefore, the observed associations should be interpreted as reflecting tumor-associated systemic inflammation rather than direct evidence of hypoxia activation.

In contrast, conventional serum tumor markers demonstrated weaker and less consistent clinicopathological correlations. CA19-9 levels showed an association with increasing age (*p* < 0.001), while CA125 exhibited a limited relationship with gross tumor morphology (*p* = 0.027). AFP displayed modest variation according to tumor location (*p* = 0.039). Notably, carcinoembryonic antigen (CEA) did not show significant associations with most clinicopathological parameters (all *p* > 0.05). Sex-related differences were minimal overall, although slightly higher LMR values were observed among female patients (*p* = 0.040). Neither inflammatory indices nor classical tumor markers demonstrated consistent relationships with vascular or perineural invasion, aside from isolated statistical findings without a uniform pattern.

Taken together, these results indicate that systemic inflammatory indices, particularly NLR, PLR, and LMR are more closely aligned with clinical indicators of tumor burden and progression than traditional serum antigens. Rather than representing specific molecular pathways, these indices likely capture the cumulative interaction between tumor growth and host immune response. Consequently, inflammatory markers provide complementary clinical information to molecular biomarkers within the multimodal diagnostic framework evaluated in this study, whereas classical tumor markers alone show limited ability to reflect underlying disease dynamics.

Positive detection rates of biomarkers in colorectal cancer patients (*n* = 142): Detection was assessed using hospital defined cutoffs for serum markers (CEA ≥ 5 ng/mL, CA19-9 ≥ 30 U/mL, CA125 ≥ 24 U/mL, AFP ≥ 7 ng/mL), and optimal thresholds were determined based on ROC analyses for Plasma mSEPT9 (≥10.01%) and Urinary DiAcSpm (≥32.32 ng/mL). Bars represent the proportion of patients testing positive, with 95% binomial confidence intervals. The dashed line indicates the 50% reference threshold. Asterisks indicate significant deviation from this benchmark by two-sided binomial testing (* *p* < 0.05, ** *p* < 0.01; *** *p* < 0.001).

### 3.7. Positive Detection Rates Across Conventional Biomarkers, mSEPT9, and DiAcSpm

**Figure 5:**
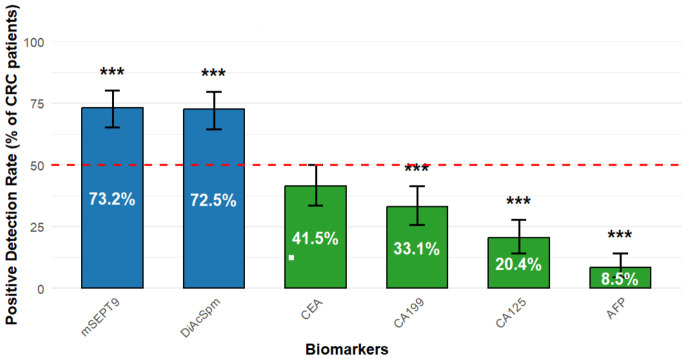
Positive detection rates of biomarkers in colorectal cancer patients. The dashed red horizontal line at 50% serves as a reference threshold to distinguish biomarkers that detect more than half of CRC cases (above the line) from those that detect less than half (below the line). It is not a statistical cutoff but a heuristic indicator of clinical utility. Biomarkers above the line (mSEPT9 at 73.2%, DiAcSpm at 72.5%) are considered clinically useful for detection. Asterisks (***) above the mSEPT9 and DiAcSpm bars indicate that their detection rates are statistically significantly different from the 50% reference line (binomial test, *p* < 0.001). For classical serum tumor markers CA19-9 (33.1%), CA125 (20.4%), and AFP (8.5%), the asterisks indicate that their detection rates are significantly worse than 50%. The white square fill pattern identifies the CEA bar and distinguishes it from the other classical markers (CA19-9, CA125, AFP). CEA (41.5%) is below 50% but is closer to the reference line; its detection rate is not significantly different from 50%, hence no asterisks are shown. Different bar fill patterns distinguish the biomarkers: experimental markers mSEPT9 and DiAcSpm are shown with solid blue fill, while classical serum tumor markers CEA, CA19-9, CA125, and AFP are shown with solid green fill. No other symbols or colors require explanation.

Comparative analyses of biomarker positivity further highlighted the diagnostic advantage of these markers (Figure 5). Plasma mSEPT9 and urinary DiAcSpm demonstrated the highest detection rates among CRC patients (73.2% and 72.5%, respectively), both significantly exceeding the 50% reference threshold (binomial test, *p* < 0.001). In contrast, classical serum markers exhibited substantially lower sensitivities, including CEA (41.5%), CA19-9 (33.1%), CA125 (20.4%), and AFP (8.5%). These results underscore the superior sensitivity of mSEPT9 and DiAcSpm, consistent with previously reported (in the literature) epigenetic and metabolic dysregulation associated with hypoxia. Compared with traditional serum antigens, these pathways appear to capture core biological features of colorectal tumors that have been associated with hypoxia in prior assessments.

### 3.8. Pairwise Comparison of Biomarkers for Assessing Detection Performance for Colorectal Cancer Using McNemar’s Test

**Figure 6:**
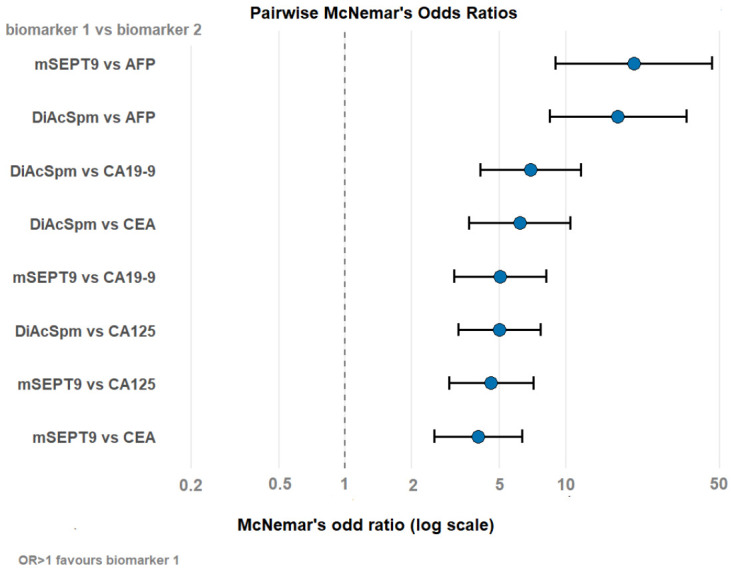
Pairwise McNemar’s odds ratios comparing novel and conventional biomarkers in colorectal cancer. The vertical dashed line at OR = 1 represents the line of no difference. Odds ratios to the right of this line (OR > 1) indicate that biomarker 1 detected significantly more CRC cases than biomarker 2. Odds ratios to the left (OR < 1) indicate that biomarker 2 was superior. Blue circles represent the odds ratios for each pairwise comparison. The x-axis uses a logarithmic scale to symmetrically display odds ratios below and above 1.

Pairwise comparisons of diagnostic performance were performed using McNemar’s test, which evaluates differences between correlated binary outcomes obtained from the same patients. This approach determines whether one biomarker detects significantly more colorectal cancer (CRC) cases than another while accounting for within-subject dependence. Results are summarized in Figure 6 and Table S5.

In McNemar analysis, n10 represents patients positive for Biomarker 1 but negative for Biomarker 2, whereas n01 represents patients positive for Biomarker 2 but negative for Biomarker 1. Odds ratios (ORs), therefore, quantify the relative detection advantage between paired tests.

Plasma methylated SEPT9 (mSEPT9) identified significantly more CRC cases than all conventional serum markers, including CEA, CA19-9, CA125, and AFP (OR range: 4.00–20.33; all adjusted *p* < 0.001). Similarly, urinary N^1^, N^12^-diacetylspermine (DiAcSpm) detected substantially more cases than these classical antigens (OR range: 5.00–17.25; all adjusted *p* < 0.001). These findings indicate markedly higher diagnostic sensitivity of the molecular and metabolic biomarkers compared with traditional antigen-based assays.

Direct comparison between mSEPT9 and DiAcSpm showed no significant difference in detection performance (OR = 0.77, 95% CI: 0.53–1.13, FDR-adjusted *p* = 0.226), suggesting that both biomarkers provide comparable diagnostic yield. The absence of superiority between them supports the interpretation that they capture partially independent biological signals rather than redundant information.

Among conventional markers, only CEA demonstrated modest superiority over AFP (OR = 3.76, adjusted *p* < 0.001), whereas most comparisons among CA19-9, CA125, and AFP were not significant, indicating substantial overlap and limited discriminatory differentiation within this group.

Overall, paired diagnostic analysis demonstrates that mSEPT9 and DiAcSpm consistently outperform conventional serum antigens in CRC detection. The complementary detection patterns observed here support combining molecular, metabolic, and conventional markers within a multi-parameter non-invasive diagnostic strategy linked to hypoxia signaling, as inferred from previous studies.

Pairwise McNemar’s odds ratios comparing novel and conventional biomarkers in colorectal cancer (CRC) patients (*n* = 142): Forest plots show odds ratios (OR) with 95% confidence intervals for discordant positive detection between biomarkers. Odds ratios are plotted on a logarithmic scale; values greater than 1 indicate that Biomarker 1 listed on the left was more frequently positive than its comparator. Plasma mSEPT9 and urinary DiAcSpm) demonstrated markedly higher positivity compared with conventional serum markers (CEA, CA19-9, CA125, and AFP). Significance was assessed by exact McNemar’s test with Benjamini–Hochberg false discovery rate (FDR) correction (*** *p* < 0.001, ** *p* < 0.01, * *p* < 0.05). Exact discordant counts are shown in Table S5 (n10 = Biomarker 1 positive only; n01 = Biomarker 2 positive only).

### 3.9. ROC Analysis and Diagnostic Performance of Individual and Multimarker Panels

Diagnostic performance was evaluated using receiver operating characteristic (ROC) curve analysis. Areas under the curve (AUCs) were compared using the DeLong test for correlated ROC curves. Sensitivity, specificity, positive predictive value (PPV), and negative predictive value (NPV) were calculated at optimal thresholds determined by the Youden index.

### 3.10. Performance of Individual Biomarkers

For all ROC analyses presented here, the non-CRC group consisted of both colorectal polyp patients and non-malignant controls (total *n* = 240).

**Figure 7:**
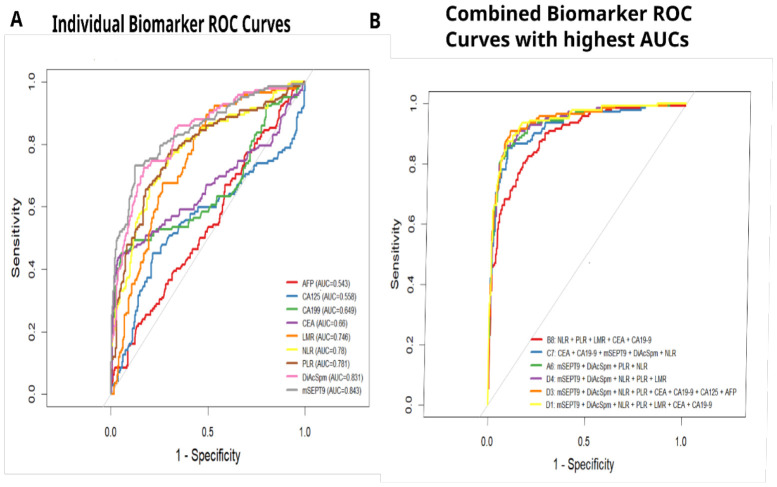
Receiver operating characteristic (ROC) analysis comparing diagnostic performance of individual biomarkers and combined multimodal models for colorectal cancer detection. Receiver operating characteristic (ROC) curve illustrating the diagnostic accuracy of individual biomarkers (**left panel**) (**A**) and combined biomarker models (**right panel**) (**B**) for discriminating colorectal cancer (CRC) from non-CRC groups. The non-CRC group includes both colorectal polyp patients (*n* = 62) and non-malignant controls (*n* = 178). (**A**) Individual biomarker performance. ROC curves show the diagnostic performance of mSEPT9; DiAcSpm; systemic indices (NLR, PLR, and LMR); and CEA, CA19-9, CA125, and AFP. The area under the curve (AUC) for each biomarker is indicated in the figure legend. (**B**) Combined biomarker models. ROC curves comparing multivariable diagnostic panels integrating molecular, metabolic, inflammatory, and classical biomarkers: B6 model: NLR + PLR + LMR + CEA + CA19-9; C7 model: CEA + CA19-9 + mSEPT9 + DiAcSpm + NLR; A6 model: mSEPT9 + DiAcSpm + PLR + NLR; D4 model: mSEPT9 + DiAcSpm + NLR + PLR + LMR; and D1 model: mSEPT9 + DiAcSpm + NLR + PLR + LMR + CEA + CA19-9. The diagonal reference line represents random classification performance (AUC = 0.50).

Among single biomarkers (Figure 7A; Table S6), plasma methylated SEPT9 demonstrated strong discriminative ability for colorectal cancer detection, achieving an AUC of 0.843 (95% CI 0.800–0.886) with a sensitivity of 73.2%. DiAcSpm showed comparable performance (AUC 0.831, 95% CI 0.788–0.874; sensitivity 72.5%). Both biomarkers substantially outperformed classical serum markers, including AFP (AUC 0.543) and CA125 (AUC 0.558). Systemic inflammatory indices exhibited intermediate diagnostic accuracy, with AUC values ranging from 0.746 to 0.781, consistent with their role as indirect indicators of tumor-associated inflammation rather than tumor-specific molecular alterations.

### 3.11. Performance of Multimarker Models

To evaluate synergistic diagnostic effects, multivariable logistic regression models integrating biomarkers across biological domains were constructed. Predicted probabilities derived from each model were used to generate ROC curves (Figure 7B; Table 5).

The highest diagnostic performance was observed in full multimarker panels (Group D), with Models D1, D3, and D4 achieving AUCs of 0.950, 0.950, and 0.947, respectively (Table 5).

Notably, Model D4 comprising mSEPT9, DiAcSpm, NLR, PLR, and LMR achieved diagnostic accuracy comparable to larger panels while excluding conventional serum antigens (CEA, CA19-9, CA125, and AFP). This streamlined composition demonstrates that combining epigenetic, metabolic, and inflammatory biomarkers provides robust discrimination without reliance on traditional tumor markers, indicating greater clinical practicality and biological coherence.

Decision curve analysis was performed to evaluate the clinical net benefit of the D4 5-marker panel compared to a simpler 2-marker model (mSEPT9 + DiAcSpm). Across a range of threshold probabilities (1–50%), the D4 panel demonstrated consistently higher net benefit than the 2-marker model, indicating that the addition of NLR, PLR, and LMR improves clinical decision-making. The D4 model also outperformed the default strategies of treating all or no patients (Supplementary Figure S1).

Because hyperplastic polyps (*n* = 6) are non-neoplastic and lack malignant potential, we performed a secondary sensitivity analysis to assess whether their inclusion in the non-CRC group diluted the panel’s ability to detect true neoplastic progression. In this analysis, we compared CRC patients (*n* = 142) with only adenomatous polyps (tubular, tubulovillous, villous, and serrated lesions; *n* = 56), excluding the six hyperplastic polyps. The D4 panel achieved an AUC of 0.926 (95% CI 0.884–0.968), which is similar to the primary AUC (0.947) (95%CI 0.924–0.970) with overlapping confidence intervals (Supplementary Table S12). This confirms that the inclusion of the small number of hyperplastic polyps (9.7% of all polyps) did not materially affect the biomarker signal for neoplastic progression.

A thorough review of an extended multimarker combination (Table S7) found that diagnostic performance improved as more biological markers were integrated. Epigenetic and metabolic marker combinations performed better than single biomarkers, with an AUC as high as 0.940 (Model A6). Models using inflammatory indices and traditional tumor markers had moderate accuracy, with a maximum AUC of 0.899 (Model B8). When classical markers were added to the epigenetic and metabolic panels, performance improved further, reaching an AUC of 0.932 (Model C7).

### 3.12. Logistic Regression Analysis

Univariate and multivariate logistic regression analyses (Table S8) showed that integrated biomarker domains were independently linked to colorectal cancer risk.

In a univariate logistic regression, age showed a strong association with colorectal cancer (CRC). People aged 70 or older had a much higher odds ratio (OR = 97.03; 95% CI: 27.87–337.85; *p* < 0.001). However, this large effect should be interpreted with caution, as most participants in the CRC group were older, which may have skewed the odds ratio. As a result, age was treated as a demographic factor rather than a direct cause of disease risk. Sex showed no significant effect. Molecular and inflammatory biomarkers were significantly associated with disease presence, with mSEPT9 and DiAcSpm demonstrating robust effects alongside elevated inflammatory ratios. Classical serum markers displayed statistically significant associations in unadjusted models; however, their odds ratios reflected cohort-related variability rather than independent diagnostic strength.

After multivariable adjustment (Table S8), mSEPT9, DiAcSpm, and the inflammatory ratios remained independently associated with CRC, whereas most classical markers lost statistical significance, indicating that their predictive value was largely explained by overlap with stronger biological signals captured by the integrated biomarker domains. Odds ratios presented in the model represent statistical associations within the study population and should be interpreted as measures of diagnostic contribution rather than causal biological effects.

### 3.13. Multivariable Risk Association Analysis for Colorectal Cancer

The standardized multivariable logistic regression analysis (Table S9) quantifies each biomarker’s contribution to colorectal cancer risk while accounting for all other variables in the optimized D4 framework. Because the biomarkers are measured in different ways, such as DNA methylation in blood, urinary metabolites, blood cell ratios, and serum proteins, all predictors were converted to Z-scores before analysis. This means the odds ratios show how the risk of colorectal cancer changes with a one-standard-deviation increase in a biomarker, making it easier to compare their effects.

**Figure 8:**
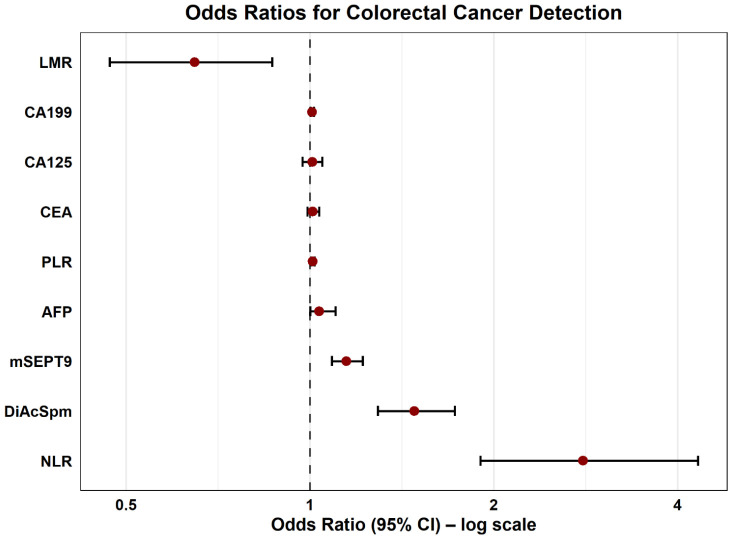
Forest plot of multivariable logistic regression for biomarker predictors of colorectal cancer. Forest plot of unadjusted odds ratios (ORs) and 95% confidence intervals for nine biomarkers in relation to colorectal cancer (CRC). ORs > 1 (right of dashed line) indicate that higher biomarker levels are associated with increased CRC risk; OR < 1 (left of dashed line) indicates a protective effect (higher LMR is associated with lower CRC risk). All D4 panel biomarkers (mSEPT9, DiAcSpm, NLR, PLR, and LMR) are statistically significant (*p* < 0.05; see Table S9). LMR is retained in the D4 model because it contributes independent predictive value and improves overall diagnostic accuracy, despite having a protective direction. Each dot (red circle) represents the odds ratio (OR) point estimate for a given biomarker. The horizontal line through each dot represents the 95% confidence interval (CI).

The forest plot (Figure 8) visually summarizes the standardized multivariable logistic regression analysis results by showing adjusted odds ratios and 95% confidence intervals centered on the reference line (OR = 1). Biomarkers to the right of this line mean higher CRC risk as their levels rise. If the intervals cross the line, it means there is no independent association after adjustment. The plot clearly separates hypoxia-related biomarkers from traditional tumor antigens. Molecular and inflammatory markers have higher odds ratios with narrower confidence intervals, while classical serum markers are close to 1, with wider confidence intervals, suggesting they contribute less when all pathways are considered together.

Of all the predictors, NLR had the strongest independent association with CRC (OR 2.80, 95% CI 1.86–4.21; *p* < 0.001). DiAcSpm (OR 1.48, 95% CI 1.28–1.71; *p* < 0.001) and mSEPT9 (OR 1.15, 95% CI 1.08–1.22; *p* < 0.001) were also significant risk factors. Notably, LMR was a protective factor (OR 0.65, 95% CI 0.48–0.88; *p* = 0.005), confirming that higher LMR reduces CRC risk. PLR shows a smaller but still significant association (OR 1.01; *p* = 0.001). Classical markers were generally non-significant after adjustment.

In contrast, classical tumor markers had little independent value. CA19-9 was statistically significant (OR 1.01; *p* = 0.007), but its effect was small, showing it has a limited impact compared to hypoxia-related biomarkers. Carcinoembryonic antigen (CEA), cancer antigen 125 (CA125), and alpha-fetoprotein (AFP) were not independently associated with CRC risk after adjustment, as their confidence intervals did not indicate a clear association, and their *p*-values were not significant. This suggests that the diagnostic value of these markers is mostly covered by the epigenetic, metabolic, and inflammatory markers in the D4 panel, which combines these biological factors.

Overall, Figure 8 and Table S9 show that the optimized model mainly distinguishes colorectal cancer risk through biological pathways related to tumor hypoxia and the body’s response, rather than traditional antigen secretion. The standardized method confirms that each part of the D4 panel adds value and provides a clear ranking of effect strength, making the results both understandable and statistically reliable. The agreement between the visual forest plot and the numerical regression results increases confidence that this multimarker approach captures distinct aspects of disease biology rather than merely repeating signals and supports its use as a clear, non-invasive diagnostic tool.

### 3.14. Internal Validation of the Integrated Diagnostic Model

To assess the potential impact of verification bias, we performed a sensitivity analysis restricted to CRC patients (*n* = 142) and the 42 control participants who underwent colonoscopy with negative findings. In this subset, the D4 panel achieved an AUC of 0.971 (95% CI 0.951–0.992), with a sensitivity of 91.5% and specificity of 92.9% at the Youden-optimized threshold. These values were similar to or slightly higher than those in the primary analysis (AUC 0.947), indicating that verification bias did not inflate diagnostic performance (Supplementary Table S12).

To address potential confounding by age and sex, we re-fitted the D4 logistic regression model including age (continuous) and sex as additional covariates. After adjustment, all biomarkers remained independently associated with CRC status (all *p* < 0.01; Supplementary Table S13). The age- and sex-adjusted AUC was 0.962 (95% CI 0.946–0.979), with a sensitivity of 94.4% and specificity of 86.2% at the Youden-optimized threshold. To assess whether the D4 panel provides diagnostic value beyond demographic factors alone, we compared the adjusted D4 model with a baseline model containing only age and sex. The baseline model achieved an AUC of 0.809 (95% CI 0.765–0.853), with a sensitivity of 74.6% and specificity of 71.7%. The adjusted D4 model significantly outperformed the baseline (DeLong test *p* < 0.0001). Similar results were observed for the other combined models (D1, D3, C7, A6, and B8), with each showing a significant increase in AUC compared to the baseline model (all *p* < 0.0001; Supplementary Table S14). Although several panels showed high performance, the D4 model was selected as the preferred panel because it uses only five readily available biomarkers (mSEPT9, DiAcSpm, NLR, PLR, and LMR), fewer than D1, D3, or C7, while achieving an adjusted AUC (0.962) comparable to the larger panels (0.965 for D1/D3). This streamlined composition enhances clinical practicality, reduces assay costs, and simplifies interpretation without sacrificing diagnostic accuracy. The age- and sex-adjusted odds ratios for all biomarkers are presented in Supplementary Table S16; the D4 panel biomarkers remained statistically significant independent predictors of CRC (*p* < 0.05).

To further address potential confounding, we performed propensity score matching (1:1 nearest neighbor matching on age and exact matching on sex) to compare CRC patients (*n* = 142) with non-cancer controls (hernia and hemorrhoids, *n* = 178). After matching, 78 CRC patients and 78 controls with closely balanced age (mean 59.8 vs. 59.0 years) and identical sex distribution were retained. In this matched subset, the D4 panel achieved an AUC of 0.967 (95% CI 0.943–0.990), with a sensitivity of 87.2% and specificity of 94.9% (Supplementary Table S15). Propensity score matching (1:1 nearest neighbor, exact on sex, caliper of 0.2 on the propensity score) retained 78 well-balanced pairs (mean age 59.8 vs. 59.0 years; sex perfectly matched). Unmatched CRC patients (*n* = 64) and controls (*n* = 100) were excluded to achieve balance, which is a standard practice in matching analyses. These results confirm that the diagnostic performance of the D4 panel is not attributable to age or sex imbalances.

Calibration of the D4 model was assessed using bootstrap resampling (1000 iterations). The calibration plot (Supplementary Figure S2) shows that the bootstrap-corrected calibration curve (solid red) lies close to the ideal 45° line (grey dashed), with a narrow 95% confidence band (grey shaded). The apparent calibration curve (solid blue) is slightly more optimistic. The Brier score was 0.081, and the apparent calibration slope and intercept were 1.00 and 0.00, respectively. These results indicate excellent agreement between predicted and observed CRC probabilities, with minimal evidence of overfitting. The logistic regression coefficients for the final D4 panel (raw units) and the predicted probability formula are presented in Supplementary Table S17.

### 3.15. Delong’s Test and McNemar’s Test for Comparison of Optimized Multimarker Panels

We directly compared the top-performing models (D1, D3, and D4) using paired DeLong and McNemar tests (Table S10). The DeLong analysis found no statistically significant differences in AUC between the panels (all *p* > 0.05), showing that their overall discrimination was similar. The McNemar test also showed no significant differences in sensitivity or specificity. These results show that the evaluated models performed similarly.

Although multiple multimarker panels demonstrated high diagnostic performance, the D4 model achieved comparable accuracy using fewer biomarkers, indicating greater clinical practicality and biological coherence.

### 3.16. Subgroup Performance of the Optimized D4 Multimarker Model to Determine Robustness

**Figure 9:**
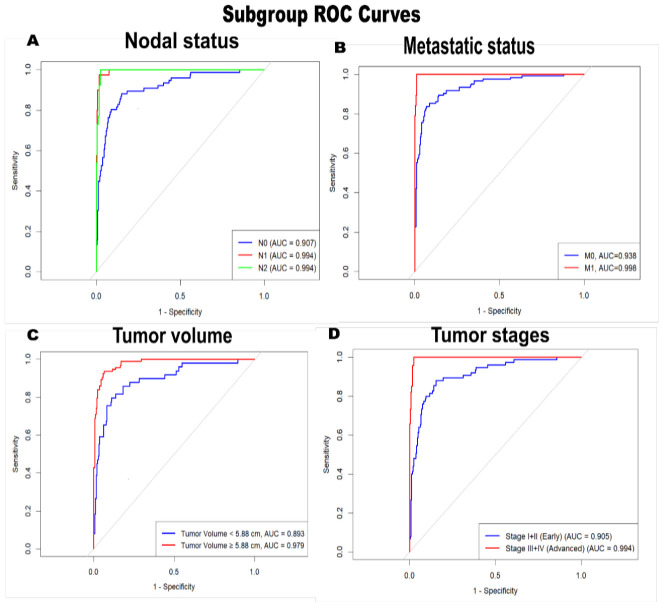
Diagnostic performance of the D4 multimarker panel across clinicopathological subgroups of colorectal cancer. Receiver operating characteristic (ROC) analyses of subgroup biomarker models. Subgroup ROC analyses of the D4 panel according to (**A**) nodal status (N0 vs. N+), (**B**) metastasis (M0 vs. M1), (**C**) tumor volume (<5.88 vs. ≥5.88 cm^3^), and (**D**) stage (early vs. advanced). The diagonal line represents the reference of no discrimination (AUC = 0.5). Sensitivity is plotted against 1–specificity.

Subgroup analyses showed that the D4 multimarker panel remained robust and stable across key colorectal cancer subgroups (Figure 9A–D; Table S11). The model performed well in early-stage disease (stages I–II; AUC = 0.905) and was highly accurate in advanced stages (stages III–IV; AUC = 0.994), indicating consistent sensitivity across tumor progression. The panel also maintained high accuracy across nodal involvement, with AUC values ranging from 0.907 in node-negative patients to 0.994 in node-positive cases. For metastatic cases, the model classified patients very accurately (M1; AUC = 0.998).

Analysis of tumor burden also showed the model’s stability. Diagnostic accuracy was higher for larger tumors (≥5.88 cm; AUC = 0.979) than for smaller ones (AUC = 0.893). When comparing age groups, D4 performed better than the more complex D3 model in younger patients (AUC 0.981 vs. 0.960; *p* = 0.002), and its performance was similar to the expanded D1 model. For patients with larger tumors, D4 clearly outperformed D1 (AUC 0.979 vs. 0.892; *p* = 0.002), showing that it is more efficient even though it is less complex.

Overall, these results show that D4 matches or exceeds the performance of larger multimarker panels, even though it uses fewer, more targeted biomarkers. Its consistent results across disease stage, tumor burden, and metastatic status support the model’s robustness. This suggests that combining epigenetic, metabolic, and systemic inflammatory signals offers a strong approach for non-invasive colorectal cancer detection.

## 4. Discussion

Colorectal cancer (CRC) is the second most common cause of cancer-related death worldwide, with the biggest increases expected in low- and middle-income regions where colonoscopy and stool-based screening are less available [79]. Although liquid biopsy methods have improved, many biomarkers still do not detect early disease well or provide sufficient biological information. In this prospective study, we found that a multimarker panel (D4) integrating molecular and systemic processes linked to tumor growth offers high diagnostic accuracy and is practical for clinical use. The main finding is that combining epigenetic (mSEPT9), metabolic (DiAcSpm), and inflammatory (NLR, PLR, and LMR) biomarkers in one test improves CRC detection compared with single markers. The D4 model showed strong diagnostic performance while remaining simple, making it a promising option for diagnostic use in patients with clinical suspicion of CRC—not for population screening.

One important limitation is that we did not directly measure tumor hypoxia—for example, by immunohistochemistry for HIF-1α/HIF-2α, pimonidazole staining, or a validated hypoxia-related gene expression signature in tumor tissue. Although we selected our biomarker panel based on published links between hypoxia and changes in epigenetic regulation, polyamine metabolism, and inflammation [15,21,26,39,60,61,78,80], we did not confirm that these markers were specifically increased by hypoxia in our patients’ tumors. Therefore, the panel should be interpreted as an empirical combination of epigenetic, metabolic, and inflammatory markers, not as a direct measure of tumor hypoxia. The biological models in Figure 1 and Figure 2 are based on the prior literature and explain why we chose these biomarkers; they do not present conclusions from our own data. Future studies should include direct tests for tumor hypoxia (e.g., HIF expression analysis or hypoxia-specific imaging) to confirm these pathways.

In our cohort of 382 participants, both plasma mSEPT9 and urinary DiAcSpm detected >70% of CRC cases and outperformed standard serum tumor markers. mSEPT9 positivity increased with higher TNM stage, greater tumor burden, and poorer differentiation, supporting its use as a marker of tumor-related epigenetic changes and disease activity. DiAcSpm levels also increased with tumor stage and depth of invasion but were less strongly associated with lymph node or metastatic status, consistent with its role as a marker of polyamine metabolism changes during tumor growth [81]. These findings suggest that mSEPT9 and DiAcSpm reflect distinct biological processes rather than redundant information.

Systemic inflammatory indices provided additional biological insight. Higher NLR and PLR were closely associated with more advanced disease, while higher LMR (indicating better immune status) was associated with lower CRC risk (protective effect). Patients who tested positive for mSEPT9 or DiAcSpm also had higher NLR and PLR, and lower LMR, suggesting that tumor-derived molecular signals correlate with systemic immune imbalance [82]. These results support a link between tumor progression, metabolic/epigenetic changes, and the host inflammatory response.

Combining these marker types in the D4 panel (mSEPT9, DiAcSpm, NLR, PLR, and LMR) yielded an AUC of 0.947, sensitivity 85.9%, and specificity 92.9%. Adding conventional tumor markers (CEA and CA19-9) did not significantly improve performance (DeLong and McNemar tests, all *p* > 0.05). Decision curve analysis further showed that the incremental net benefit of the full D4 panel over the simpler 2-marker model (mSEPT9 + DiAcSpm) was modest, with overlapping confidence intervals across most threshold probabilities, indicating that adding NLR, PLR, and LMR provides limited clinical utility beyond the two core biomarkers. The D4 model’s strength lies in focusing on biologically related pathways rather than simply adding more markers.

Earlier studies have shown that mSEPT9 or DiAcSpm are useful for diagnosis and have combined methylation markers with inflammatory indices [83,84,85]. Our study goes further by integrating epigenetic, metabolic, and immune-inflammatory biomarkers into a single test and demonstrating improved diagnostic performance. By including colorectal polyps (adenomas with dysplasia), the D4 panel may be relevant for detecting lesions along the adenoma–carcinoma sequence. Of note, hyperplastic polyps (non-neoplastic) were only included in the primary non-CRC group to reflect real-world clinical lesion distribution; they were not considered part of the premalignant spectrum. A sensitivity analysis restricted to adenomatous polyps (excluding hyperplastic) confirmed that the panel retains excellent performance (AUC 0.926). The biomarker results for polyp patients fell between those for CRC and non-cancer controls, supporting the panel’s ability to detect changes along this continuum.

## 5. Study Limitations

Several limitations should be considered. First, this was a single-center study; the participants may not reflect the full diversity of other regions, ethnic groups, or healthcare systems, limiting generalizability. Second, the sample size within specific subgroups (e.g., pT1 for DiAcSpm) was small, which can increase variability; larger studies are needed to confirm robustness. Third, urinary DiAcSpm concentrations were reported as absolute values (ng/mL) without creatinine normalization. While this avoids confounding by variable creatinine excretion (e.g., in older patients or those with comorbidities), it means that an artificially high reading could result from a highly concentrated urine sample (e.g., dehydration), and dilute urine could lead to falsely low levels. This pre-analytical limitation could cause false-positive results in dehydrated patients or false-negative results in overhydrated patients, which is a major concern for clinical implementation. Therefore, our reported absolute values should be interpreted with caution, and future validation must include creatinine normalization. Future studies should measure creatinine and report normalized results (e.g., ng/mg creatinine). Fourth, the age distribution was uneven (many CRC patients aged ≥70 years), which may inflate odds ratios; age-matched or stratified analyses are needed. Fifth, the model was developed and internally validated on the same dataset. Although we performed bootstrap resampling (1000 iterations) and calibration analysis, these do not replace external validation. The optimal cutoffs for mSEPT9 and DiAcSpm were determined within the same cohort using the Youden index, which can lead to overfitting and optimistic performance estimates. Therefore, the reported AUC (0.947) and other accuracy metrics may be optimistic. External validation in an independent, preferably multi-center, cohort is essential before any clinical application. Six hyperplastic polyps (non-neoplastic) were included in the primary non-CRC group, which could theoretically dilute signals for neoplastic progression. However, a sensitivity analysis comparing CRC with only adenomatous polyps (excluding hyperplastic) yielded an AUC of 0.926, similar to the primary AUC (0.947), indicating no meaningful dilution.

## 6. Clinical Implications

This study stands out because it was prospectively designed, compared new and traditional biomarkers simultaneously, and used robust statistical methods (ROC, regression, and DCA). The D4 panel requires only blood and urine samples, avoiding colonoscopy or stool tests. It is effective for early-stage disease and detects nearly all metastatic CRC cases. The required tests (PCR, ELISA, and complete blood count) are widely available, making the approach practical and potentially implementable—especially in regions with limited access to endoscopy.

## 7. Future Directions

Large, multi-center, prospective studies across diverse populations are needed to validate the D4 panel and fine-tune its cutoffs. Long-term studies should assess its utility for monitoring recurrence, treatment response, and prognosis. Economic evaluations of cost-effectiveness compared with existing screening strategies are necessary to support adoption. Incorporation of machine learning may enhance risk stratification. Finally, direct measurement of hypoxia-related signals (e.g., HIF expression and hypoxia imaging) in tumor tissue would strengthen the biological underpinning of the panel.

## 8. Conclusions

Our results show that the D4 multimarker panel is a promising non-invasive approach for CRC detection. By combining epigenetic, metabolic, and inflammatory biomarkers, the panel achieves high diagnostic accuracy and is practical for clinical use. Its strong performance in early-stage disease highlights its potential for diagnostic use in patients presenting with symptoms or risk factors. Screening applications would require prospective validation in an asymptomatic population, which was not performed here. Rather than directly measuring upstream factors such as tumor hypoxia, the panel detects downstream biological effects in blood and urine. This practical approach makes it usable in diverse healthcare settings, especially where invasive procedures (e.g., colonoscopy) are not readily available (e.g., LMICs). Nevertheless, the model requires external validation before any claim of broad clinical implementation can be made; the current findings are promising but preliminary.

## Figures and Tables

**Table 1:** Distribution of CRC and non-CRC diagnoses by sex and age group with mean age.

Diagnosis	Description	Total	Gender		Age Groups				
			Male	Female	<50	50–59	60–69	≥70	Mean
CRC	Overall	142	76	66	6	29	66	41	65
	Right Colon	38	16	22	2	6	17	13	66
	Left Colon	65	38	27	1	12	35	17	65
	Rectum	39	22	17	3	11	14	11	63
	Stage I	35	21	14	1	5	21	8	65
	Stage II	40	19	21	1	8	20	11	65
	Stage III	48	26	22	2	14	15	17	65
	Stage IV	19	10	9	2	2	10	5	66
Non-cancer subgroups									
Adenomatous Colorectal polyps		62	33	29	2	18	29	3	59
Inguinal Hernia		64	39	25	16	31	16	1	54
Hemorrhoids		114	55	59	53	36	24	1	50
Total		382	203	179	77	124	135	46	58

**Table 2:** Clinicopathological and lifestyle characteristics of colorectal cancer, colorectal polyps, and control groups.

Category	Variable	CRC *n* = 142	Polyps *n* = 62	Controls *n* = 178	*p*-Value
**Demographics**	**Sex**				0.992
	Male	76 (37.4%)	33 (16.3%)	94 (46.3%)	
	Female	66 (36.9%)	29 (16.2%)	84 (46.9%)	
**Clinical parameters**	**Tumor Gross Type**				
	Ulcerative	80 (56.3%)	—	—	
	Polypoid	40 (28.2%)	—	—	
	Unknown	22 (15.5%)	—	—	
	**Tumor Infiltration**				
	Mucosa	4 (2.8%)	—	—	
	Sub mucosa	5 (3.5%)	—	—	
	Muscularis propria	51 (35.9%)	—	—	
	Pericolic tissue	44 (31.0%)	—	—	
	Serosa	18 (12.7%)	—	—	
	Adjacent structures	20 (14.1%)	—	—	
	**Lymph Node Ratio**			—	
	<0.2 (low)	112 (78.9%)	—	—	
	≥0.2 (high)	30 (21.1%)	—	—	
	**Tumor Budding**				
	Bd1 (Low)	62 (43.7%)	—	—	
	Bd2 (Intermediate)	45 (31.7%)	—	—	
	Bd3 (High)	35 (24.6%)	—		
	**Lymph Node Invasion**			—	
	Absent	78 (54.9%)	—	—	
	Present	64 (45.1%)	—	—	
	**Vascular Invasion**				
	Absent	70 (49.3%)	—	—	
	Present	72 (50.7%)	—	—	
	**Perineural Invasion**			—	
	Absent	83 (58.5%)	—	—	
	Present	59 (41.5%)	—	—	
	**p53 Mutation Pattern**			—	
	Wild-type	48 (33.8%)	—		
	Mutant pattern	76 (53.5%)	—	—	
	Indeterminate	18 (12.7%)	—	—	
	**Ki-67 index group** **(median-IQR)**	60 (50–70)	—	—	
	**Colorectal Polyps**				
	Tubular adenoma	—	23 (37.1%)	—	
	Tubulovillous adenoma	—	17 (27.4%)	—	
	Villous adenoma	—	11 (17.7%)	—	
	Serrated lesions	—	5 (8.1%)	—	
	**Hyperplastic polyps**	—	6 (9.7%)	—	
	**Colorectal Polyps Dysplasia Grade**				
	Low-grade dysplasia		22(35.5%)		
	High-grade dysplasia		40(64.5%)		
**Lifestyle parameters**	**Smoking History**				0.265
	Never smoked	54 (38.0%)	31 (50.0%)	—	
	Former smoker	68 (47.9%)	23 (37.1%)	—	
	Current smoker	20 (14.1%)	8 (12.9%)	—	
	**Alcohol History**				0.007 *
	Never drank	52 (36.6%)	24 (38.7%)	—	
	Former drinker	72 (50.7%)	20 (32.3%)	—	
	Current drinker	18 (12.7%)	18 (29.0%)	—	
	**Family History of Cancer**				
	No	55 (38.7%)	21 (34.4%)	—	0.811
	Yes	24 (16.9%)	12 (19.7%)	—	
	Unknown	63 (44.4%)	28 (45.9%)	—	

Hyperplastic polyps (*n* = 6) are non-neoplastic but were included to represent real-world lesion distribution. Excluding all polyps from the analysis did not materially change the diagnostic performance (see the sensitivity analysis in the Results). Data are presented as *n* (%) for categorical variables and median (IQR) for continuous variables. *p*-values were obtained using Chi-square or Fisher’s exact test for categorical variables and Mann–Whitney U test for continuous variables, where appropriate. Significance was set at *p* < 0.05. Lymph node ratio (LNR) = number of positive lymph nodes/number of examined lymph nodes; cutoff of ≥0.20 selected based on published prognostic studies in colorectal cancer (Berger et al., *J Clin Oncol* 2005; Park *Ann Coloproctol* 2013 [72,73]). *p* < 0.05. Data are presented as *n* (%). *p*-values were calculated using the Chi-square test or Fisher’s exact test, as appropriate. * indicates *p* < 0.05 (statistically significant).

**Table 3:** McNemar’s test comparison of paired diagnostic performance between biomarkers across diagnostic categories and clinicopathological stages.

McNemar’s *p*-Value for Diagnostic Groups	McNemar’s *p*-Value for Stages	McNemar’s *p*-Value for pT Stage	McNemar’s *p*-Value for Lymph Node Stage
CRC (*p* = 0.894)	Stage I (*p* = 0.050 *)	pT1 (*p* = 0.250)	N0 (*p* = 0.596)
Polyps (*p* = 0.570)	Stage II (*p* = 0.450)	pT2 (*p* = 0.061)	N1 (*p* = 0.724)
Non-Malignant (*p* = 0.031 *)	Stage III (*p* = 0.340)	pT3 (*p* = 0.0197 *)	N2 (*p* = 0.450)
-	Stage IV (*p* = 0.620)	pT4 (*p* = 1.00)	-

* indicates *p* < 0.05 (statistically significant).

**Table 4:** Pairwise comparison of mSEPT9 and DiAcSpm positivity and negativity status in correlation with inflammatory markers.

Marker	mSEPT9-Positive	mSEPT9-Negative	DiAcSpm-Positive	DiAcSpm-Negative	*p*-Value
NLR	3.05 (2.23–3.86)	2.20 (1.78–2.23)	2.83 (2.12–3.69)	2.24 (1.81–2.96)	<0.001 **
PLR	181.25 (137.79–228.96)	126.19 (107.38–166.02)	174.26 (123.51–232.65)	128.19 (108.06–168.49)	<0.001 **
LMR	2.49 (1.92–3.28)	3.50 (2.66–4.89)	2.65 (1.93–3.42)	3.43 (2.60–4.88)	<0.001 **

*p* values derived from Mann–Whitney U test comparing mSEPT9-positive vs. mSEPT9-negative patients. *p* values derived from Mann–Whitney U test comparing DiAcSpm-positive vs. DiAcSpm-negative patients. Statistical significance was set at *p* < 0.05. Data presented as median (interquartile range). ** indicates *p* < 0.001 (statistically significant).

**Table 5:** Receiver operating characteristic (ROC) analyses for combined biomarker models with the highest area under the curve (AUC).

Model Group	Biomarker Combination	AUC (95% CI)	Sensitivity (%)	Specificity (%)	*p*-Value
D4	**mSEPT9 + DiAcSpm + NLR + PLR + LMR**	**0.947 (0.924–0.970)**	**85.9**	**92.9**	<0.001 **
**D1**	mSEPT9 + DiAcSpm + NLR + PLR + LMR + CEA + CA19-9	0.950 (0.928–0.973)	86.6	92.5	<0.001 **
**D3**	mSEPT9 + DiAcSpm + NLR + PLR + CEA + CA19-9 + CA125 + AFP	0.950 (0.927–0.973)	90.8	90.4	<0.001 **
**C7**	CEA + CA19-9 + mSEPT9 + DiAcSpm + NLR	0.932 (0.904–0.959)	85.2	91.7	<0.001 **
**A6**	mSEPT9 + DiAcSpm + PLR + NLR	0.940 (0.918–0.967)	85.2	92.5	<0.001 **
**B8**	NLR + PLR + LMR + CEA + CA19-9	0.899 (0.867–0.932)	93.2	80.3	<0.001 **

For all ROC analyses in this table, the comparator (non-CRC) group consists of colorectal polyp patients (*n* = 62) and non-malignant controls (hernia and hemorrhoid patients, *n* = 178). CRC = colorectal cancer. AUC = area under the curve. CI = confidence interval. ** indicates *p* < 0.001(statistically significant), meaning the combined biomarker model is statistically significantly better than random chance (null hypothesis AUC = 0.50).

## Data Availability

The original contributions presented in this study are included in the article/Supplementary Material. Further inquiries can be directed to the corresponding author.

## Supplementary Materials

The following supporting information can be downloaded at https://www.mdpi.com/article/10.3390/diagnostics16121753/s1. Table S1. Comparison of clinicopathological features between mSEPT9-positive (≥10.01%) and mSEPT9-negative (<10.01%) colorectal cancer patients (*n* = 382). Table S2. Comparison of clinicopathological features between DiAcSpm-positive (≥32.32 ng/mL) and DiAcSpm-negative (<32.32 ng/mL) colorectal cancer patients (*n* = 382). Table S3. Comparative distributions of epigenetic (mSEPT9), metabolic (DiAcSpm), inflammatory (NLR, PLR, LMR), and classical serum biomarkers (CEA, CA19-9, CA125, AFP) across diagnostic groups: colorectal cancer (CRC, *n* = 142), colorectal polyps (*n* = 62), and non-cancer controls (*n* = 178). Table S4. Associations of classical serum tumor markers (CEA, CA19-9, CA125, AFP) and systemic inflammatory indices (NLR, PLR, LMR) with clinicopathological characteristics in colorectal cancer patients (*n* = 142). Table S5. Pairwise McNemar’s test comparing plasma methylated SEPT9 (mSEPT9), urinary N^1^,N^12^-diacetylspermine (DiAcSpm), and classical serum markers (CEA, CA19-9, CA125, AFP) in the same cohort of 382 participants. Table S6: Receiver operating characteristic (ROC) analysis comparing plasma epigenetic (mSEPT9), urinary metabolic (N^1^,N^12^-diacetylspermine, DiAcSpm), systemic inflammatory (NLR, PLR, LMR), and classical serum (CEA, CA19-9, CA125, AFP) biomarkers for distinguishing colorectal cancer (CRC) from non-CRC individuals. Table S7: Evaluation of multimarker combinations integrating plasma epigenetic (mSEPT9), urinary metabolic (N^1^,N^12^-diacetylspermine, DiAcSpm), systemic inflammatory (NLR, PLR, LMR), and classical serum (CEA, CA19-9, CA125, AFP) biomarkers for distinguishing colorectal cancer (CRC) from non-CRC individuals. Table S8. Binary logistic regression models examining associations with colorectal cancer (CRC) status (1 = CRC, 0 = non-CRC). Table S9. Multivariable logistic regression model including all nine biomarkers simultaneously (mSEPT9, DiAcSpm, NLR, PLR, LMR, CEA, CA19-9, CA125, AFP) without adjustment for age or sex. Table S10. Comparison of the three best-performing multimarker panels (D1, D3, and D4) for distinguishing colorectal cancer (CRC) from non-CRC individuals. Table S11. Performance of three optimized biomarker panels (D1, D3, and D4) stratified by clinicopathological variables: age (<60 vs. ≥60 years), tumor volume (<5.88 vs. ≥5.88 cm^3^), tumor stage (early: I + II vs. advanced: III + IV), N stage (N0, N1, N2), M stage (M0 vs. M1), and lymph node invasion (absent vs. present). Table S12. Performance of the D4 multimarker panel (mSEPT9 + DiAcSpm + NLR + PLR + LMR) under three different comparator definitions. Table S13. Performance of the D4 multimarker panel (mSEPT9 + DiAcSpm +NLR + PLR + LMR) before and after adjustment for age and sex, compared with a baseline model containing only age and sex. Table S14. Performance of six multimarker models after adjustment for age (continuous) and sex, compared with a baseline logistic regression model containing only age and sex. Table S15: Comparison of the D4 panel’s diagnostic performance under three analytical approaches: primary (unadjusted), covariate adjustment, and propensity score matching. Table S16: Results from a multivariable logistic regression model that included all nine biomarkers (mSEPT9, DiAcSpm, NLR, PLR, LMR, CEA, CA19 9, CA125, AFP) plus age (continuous) and sex as covariates. Table S17. Logistic regression coefficients for the final D4 multimarker panel (mSEPT9 + DiAcSpm + NLR + PLR + LMR) fitted on the complete case dataset (*n* = 382). For Supplementary Methods File S1 (mSEPT9): ‘Detailed protocol for plasma methylated SEPT9 (mSEPT9) quantification. This file includes cfDNA extraction, bisulfite conversion, quantitative methylation-specific PCR (qMSP) conditions, primer and probe sequences (Supplementary Method Table S1), thermal cycling parameters, Ct cutoff rules, calculation of ΔCt-based methylation score, calibration curve derivation (Supplementary Method Figure S1), and assay validation. All procedures were performed according to the manufacturer’s instructions and optimized for low-abundance methylated DNA detection.’ For Supplementary Methods File S2 (DiAcSpm): Detailed protocol for urinary N^1^,N^12^-diacetylspermine (DiAcSpm) quantification. This file describes the competitive ELISA method, sample dilution, standard curve preparation (0–40 ng/mL), plate-specific calibration parameters (Supplementary Method Table S2), exponential regression model for OD vs. log_10_-transformed concentration, calculation of final concentrations (×5 dilution factor), quality control procedures (intra- and inter-assay CV < 10%), and representative calibration curves (Supplementary Method Figure S2). Boxplot distribution of DiAcSpm levels across diagnostic groups is also shown (Supplementary Method Figure S2). For Data Sheet S1 (mSEPT9 raw data): ‘Supplementary Data Sheet S1: Raw data for plasma methylated SEPT9 (mSEPT9) quantification. The file contains two sheets: ‘Results’ with sample IDs, Ct values for background (B_Ct), methylated (M) and unmethylated (U) target DNA, ΔCt (M-U), 2^ΔCt, calibrated methylation percentage, and calculated methylation score (%). The second sheet ‘Standard Curve’ contains the calibration curve parameters and calculations used to derive methylation percentages from ΔCt values. All data are from 382 participants (142 CRC, 62 polyps, 178 non-cancer controls). For Data Sheet S2 (DiAcSpm raw data): ‘Supplementary Data Sheet S2: Raw data for urinary N^1^,N^12^-diacetylspermine (DiAcSpm) quantification. The file contains three sheets; ‘Data 1’, ‘Data 2; Data 3; each corresponding to one ELISA plate. Each sheet includes raw optical density (OD) readings at 450 nm for standards (S0–S5) and participant samples, standard concentration values, log-transformed concentrations, percentage absorbance calculations, standard curve parameters (a and b) for the exponential regression model y = a•e^(bx), and calculated DiAcSpm concentrations (ng/mL) for each sample. The final concentrations were multiplied by the dilution factor (×5). Data cover all 382 participants. For Data Sheet S3 (inflammatory ratio biomarkers raw data): ‘Supplementary Data Sheet S3: Raw data for inflammatory ratio biomarkers (NLR, PLR, LMR). The file contains absolute counts for lymphocytes, neutrophils, monocytes, and platelets for each participant (*n* = 382). Calculated ratios (NLR, PLR, LMR) are provided using Excel formulas (shown as ‘= C2/B2’, etc.). Reference ranges for absolute counts are indicated in the column headers (e.g., Lymphocytes 1.1–3.2 ×10^9^/L). All data are from the same 382 participants included in the main analysis.’ Supplementary Figure S1: Decision curve analysis comparing the clinical net benefit of the D4 5-marker panel (mSEPT9 + DiAcSpm + NLR + PLR + LMR) with a simpler 2-marker model (mSEPT9 + DiAcSpm) and reference strategies (‘Treat All’, ‘Treat None’). Supplementary Figure S2: Calibration plot for the D4 colorectal cancer prediction model, based on 1000 bootstrap resamples.

## Abbreviations

**mSEPT9**	Methylated septin9 gene
**DiAcSpm**	N^1^, N^12^-diacetylspermine
**CEA**	Carcinoembryonic antigen
**CA19-9**	Carbohydrate antigen 19-9
**CA125**	Carbohydrate antigen 125
**PLT**	Platelet
**NLR**	Neutrophil–lymphocyte ratio
**PLR**	Platelet–lymphocyte ratio
**ROC**	Receiver operating characteristic
**AUC**	Area under the ROC curve
**PCR**	Polymerase chain reaction
**LC–MS/MS**	Liquid chromatography–tandem mass spectrometry
**ELISA**	Enzyme-linked immunosorbent assay
**DNMTs**	DNA methyltransferases
**ODC**	Ornithine decarboxylase
**SAT1**	Spermine N^1^-acetyltransferas
**VEGF**	Vascular endothelial growth factor
**EGFR**	Epidermal growth factor
**dcSAM**	Decarboxylated S-adenosylmethionine
**AMD1**	Adenosylmethionine decarboxylase 1
**HRP**	Horseradish peroxidase
**TMB**	(3, 3′, 5, 5′-Tetramethylbenzidine)
**HIF**	Hypoxia-inducible factor
**C-MYC**	A transcription factor that regulates genes involved in cell growth
**FIT**	Fecal immunochemical test
**HCT**	Hematocrit
