# Supplementary material for: Hypoxia Associated Integration of Epigenetic, Metabolic, and Immune Biomarkers in Blood and Urine for Early Colorectal Cancer Detection: A Multimarker Panel
Source: Diagnostics (Basel). 2026 Jun 6;16(12):1753. doi: 10.3390/diagnostics16121753 (PMC13298955; doi:10.3390/diagnostics16121753)
Supplement: Supplementary file 1 [file diagnostics-16-01753-s001.zip › Supplementary Figure S_2.pdf]

Supplementary Figure S 2

Supplementary Figure S2: Calibration plot for the D4 CRC Prediction Model

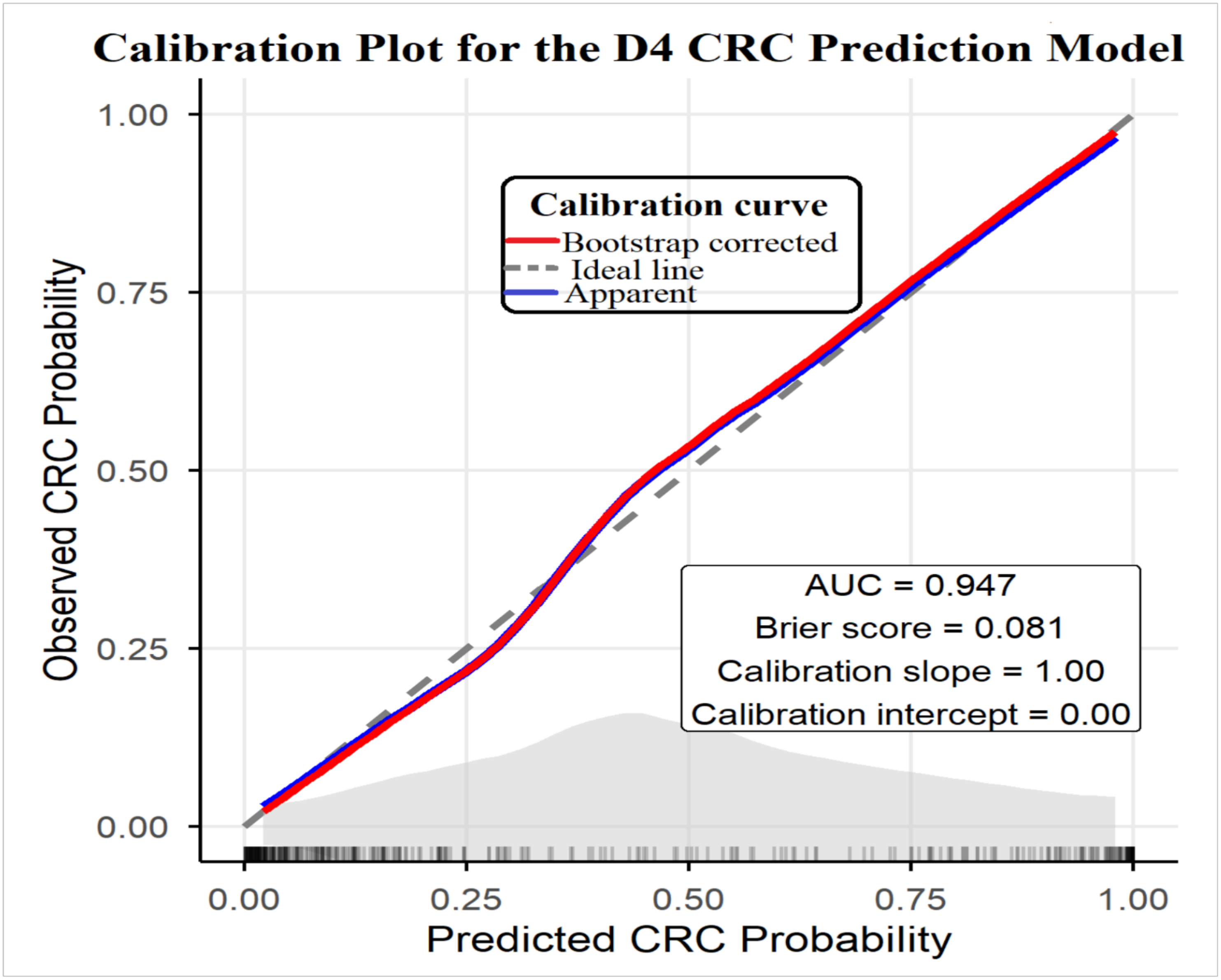

Calibration curves based on 1000 bootstrap resamples. The red curve represents the bootstrap-corrected calibration, with the grey shaded area indicating the 95% confidence band. The blue curve shows the apparent calibration (model fit on the original data). The grey dashed line is the ideal 45° line (perfect calibration). Calibration metrics (apparent) are: AUC = 0.947, Brier score = 0.081, calibration slope = 1.00, calibration intercept = 0.00. The bootstrap-corrected curve lies close to the ideal line, indicating good overall calibration and minimal overfitting
