# Supplementary material for: Hypoxia Associated Integration of Epigenetic, Metabolic, and Immune Biomarkers in Blood and Urine for Early Colorectal Cancer Detection: A Multimarker Panel
Source: Diagnostics (Basel). 2026 Jun 6;16(12):1753. doi: 10.3390/diagnostics16121753 (PMC13298955; doi:10.3390/diagnostics16121753)
Supplement: Supplementary file 1 [file diagnostics-16-01753-s001.zip › Supplementary Figure_S1.pdf]

Supplementary Figure S1**:** Decision Curve Analysis for Colorectal Cancer Prediction Models D4 5-marker panel with a simpler 2-marker model (mSEPT9 + DiAcSpm).

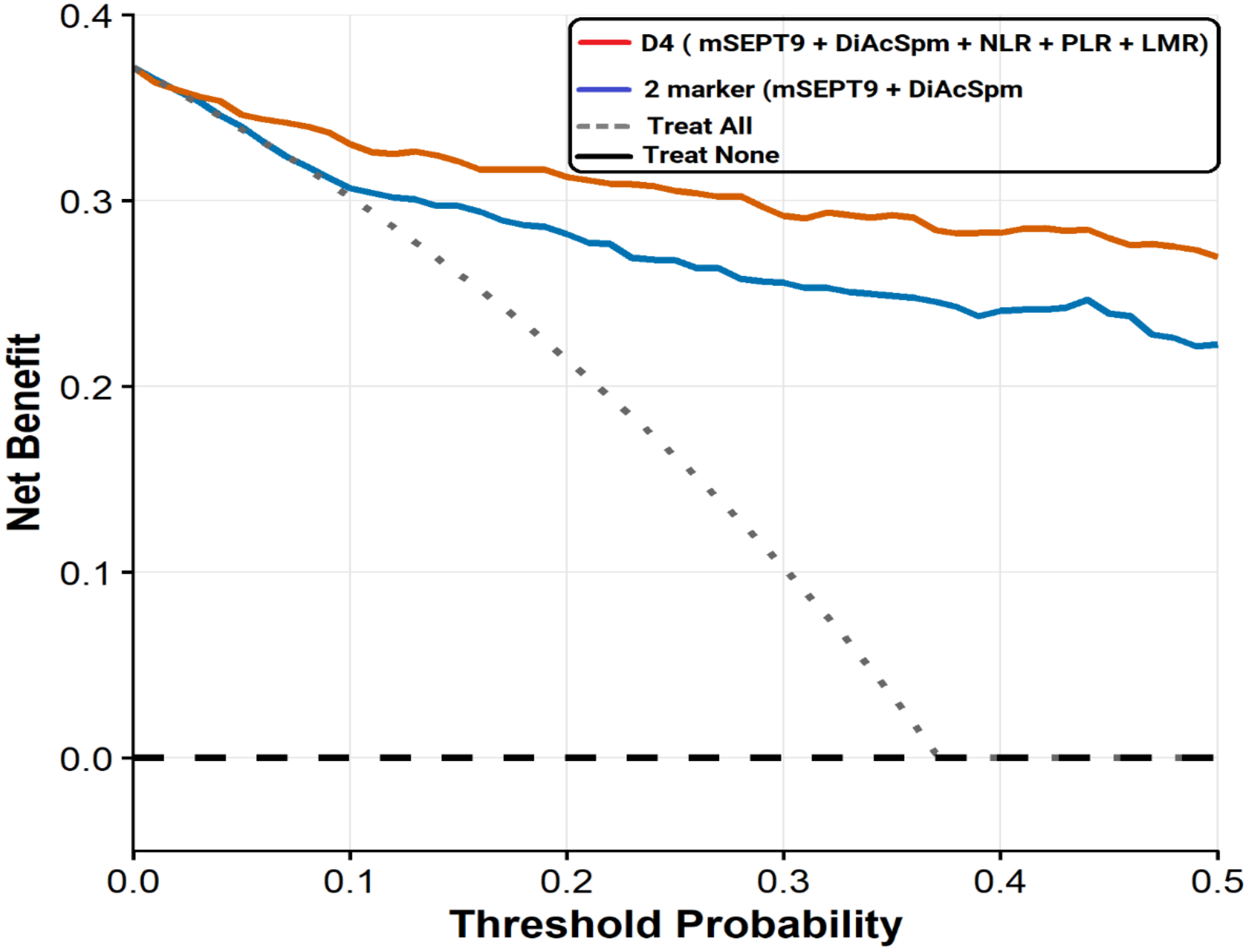

The x-axis shows the threshold probability (the risk level at which a clinician would consider a patient high-risk and act). The y-axis is net benefit, a measure that weights true positives against false positives. The red solid line represents the D4 5-marker panel (mSEPT9, DiAcSpm, NLR, PLR, LMR); the blue solid line represents the 2-marker model (mSEPT9 + DiAcSpm). The grey dotted line ("Treat All") and black dashed line ("Treat None") are reference strategies. Higher net benefit indicates better clinical utility. The D4 panel shows superior net benefit across the clinically relevant threshold range (1-50%), supporting the added value of NLR, PLR, and LMR.
