# Supplementary material for: Hypoxia Associated Integration of Epigenetic, Metabolic, and Immune Biomarkers in Blood and Urine for Early Colorectal Cancer Detection: A Multimarker Panel
Source: Diagnostics (Basel). 2026 Jun 6;16(12):1753. doi: 10.3390/diagnostics16121753 (PMC13298955; doi:10.3390/diagnostics16121753)
Supplement: Supplementary file 1 [file diagnostics-16-01753-s001.zip › Supplementary_ Table_S1.pdf]

Table S1: Comparison of Clinicopathological between mSEPT9 positive (≥10.01%) and mSEPT9-negative(<10.01%) colorectal cancer patients.

Parameters	Overall	mSEPT9-positive≥10.01%	mSEPT9-negative <10.01%	P-Value
**Number**	382	134(35.1)	248(64.9)	
**Sex**				0.366
Male	203	67(33.0)	136(67.0)	
Female	179	67(37.4)	112(62.6)	
**Age**				<0.001**
<60	204	43(21.1)	161(78.9)	
≥60	178	91(51.1)	87(48.9)	
**Tumor Volume**				
<5.88	49	25(51.0)	24(49.0)	<0.001**
≥5.88	93	79(84.9)	14(15.1)	
**Location**				0.472
Right Colon	38	30(78.9)	8(21.1)	
Left Colon	65	48(73.8)	17(26.2)	
Rectum	39	26(66.7)	13(33.3)	
**Gross Type**				0.370
Ulcerative	80	62(77.5)	18(22.5)	
Polypoid	40	28(70.0)	12(30.0)	
Unknown	22	14(63.6)	8(36.4)	
**Differentiation**				0.042*
Low	11	8(72.7)	3(27.3)	
Low-Moderate	55	34(61.8)	21(38.2)	
Moderate	52	40(76.9)	12(23.1)	
High	24	22(91.7)	2(8.3)	
**Tumor stage**				<0.001**
Stage I	35	12(34.3)	23(65.7)	
Stage II	40	29(72.5)	11(27.5)	
Stage III	48	45(93.8)	3(6.3)	
Stage IV	19	18(94.7)	1(5.2)	
**T stage**				<0.001**
T1	6	3(50)	3(50)	
T2	29	9(31)	20(69)	
T3	77	66(85.7)	11(14.3)	
T4	30	26(86.7)	4(13.3)	
**N stage**				<0.001**
N0	76	45(59.2)	31(40.8)	
N1	40	35(87.5)	5(12.5)	
N2	26	24(92.3)	2(7.7)	
**M stage**				0.023*
M0	123	86(69.1)	37(30.9)	
M1	19	1(5.3)	18(94.7)	

**Lymph node invasion**				<0.001**
Absent	78	46(41)	32(59)	
Present	64	58(90.6)	6(9.4)	
**Vascular invasion**				0.106
Absent	70	47(67.1)	23(39.1)	
Present	72	57(79.2)	15(20.8)	
**Perineural invasion**				0.065
Absent	83	56(67.5)	27(32.5)	
Present	59	48(81.4)	11(18.6)	

Table S1. Comparison of clinicopathological features between mSEPT9-positive (≥10.01%) and mSEPT9-negative (<10.01%) colorectal cancer patients (n = 382). Values are presented as subgroup frequencies with percentages in parentheses. Statistical significance was assessed using the χ^2 test or Fisher's exact test, as appropriate.

Abbreviations: mSEPT9, methylated septin 9; T stage, tumor invasion depth; N stage, lymph node involvement; M stage, distant metastasis.

Interpretation: mSEPT9 positivity was significantly associated with advanced T stage, N stage, M stage, larger tumor volume (≥5.88 cm^3), and poorer tumor differentiation ($p < 0.05$ for all). No significant associations were observed with sex, tumor location, gross tumor type, vascular invasion, or perineural invasion.

Significance levels: *$p < 0.05$; **$p < 0.01$.
