# Supplementary material for: Hypoxia Associated Integration of Epigenetic, Metabolic, and Immune Biomarkers in Blood and Urine for Early Colorectal Cancer Detection: A Multimarker Panel
Source: Diagnostics (Basel). 2026 Jun 6;16(12):1753. doi: 10.3390/diagnostics16121753 (PMC13298955; doi:10.3390/diagnostics16121753)
Supplement: Supplementary file 1 [file diagnostics-16-01753-s001.zip › Supplementary_ Table_S10.pdf]

Table S10. Head-to-head comparison of top-performing multimarker panels for colorectal cancer detection using Youden-optimised thresholds.

| Model | AUC (95% CI) | Sensitivity (%) | Specificity (%) | DeLong p vs D1 | DeLong p vs D3 | DeLong p vs D4 | McNemar p vs D1 | McNemar p vs D3 | McNemar p vs D4 |
|---|---|---|---|---|---|---|---|---|---|
| D1 | 0.950 (0.928–0.973) | 86.6 | 92.5 | – | 0.9303 | 0.1033 | – | 0.0098 | 0.6171 |
| D3 | 0.950 (0.927–0.973) | 90.8 | 90.4 | 0.9303 | – | 0.5902 | 0.0098 | – | 0.0036 |
| D4 | 0.947 (0.924–0.970) | 85.9 | 92.9 | 0.1033 | 0.5902 | – | 0.6171 | 0.0036 | – |

Table S10. Comparison of the three best-performing multimarker panels (D1, D3, and D4) for distinguishing colorectal cancer (CRC) from non-CRC individuals. All analyses used the same non-CRC comparator group (colorectal polyps, n = 62, plus non-malignant controls, n = 178; total non-CRC = 240).

Models:

- D1: mSEPT9 + DiAcSpm + NLR + PLR + LMR + CEA + CA19-9

- D3: mSEPT9 + DiAcSpm + NLR + PLR + CEA + CA19-9 + CA125 + AFP

- D4:mSEPT9 + DiAcSpm + NLR + PLR + LMR

Metrics reported:

- AUC (95% CI): Area under the ROC curve with 95% confidence interval.

- Sensitivity (%) and Specificity (%): calculated at the Youden index threshold for each model.

- DeLong p-value: comparison of AUCs between models (test of overall discrimination).

- McNemar p-value: comparison of binary classifications (CRC vs. non-CRC) at each model's optimal threshold.

Abbreviations: CRC, colorectal cancer; mSEPT9, methylated septin 9; DiAcSpm, $N^1$,$N^{12}$-diacetylspermine; NLR, neutrophil-to-lymphocyte ratio; PLR, platelet-to-lymphocyte ratio; LMR, lymphocyte-to-monocyte ratio; CEA, carcinoembryonic antigen; CA19-9, carbohydrate antigen 19-9; CA125, carbohydrate antigen 125; AFP, alpha-fetoprotein; AUC, area under the curve; CI, confidence interval.

Interpretation: DeLong tests showed no significant differences in AUC between any of the panels (all $p > 0.05$), indicating equivalent overall discrimination.

- McNemar tests showed a significant difference only between D3 and D4 ($p = 0.0036$), which is expected because D3 includes additional classical markers (CEA, CA19-9, CA125, AFP) and therefore classifies some patients differently. This does not affect the conclusion that all three panels have equivalent diagnostic accuracy (AUC).

- The D4 model metrics in this table are derived from the same dataset using the Youden-optimized cutoffs (≥10.01% for mSEPT9, ≥32.32 ng/mL for DiAcSpm); any minor differences from Table 5 are due to rounding or different comparator subgroups. Significance levels: ** $p < 0.05$ was considered statistically significant for McNemar tests; DeLong tests used $p < 0.05$ as the threshold for significance.
