# Supplementary material for: Hypoxia Associated Integration of Epigenetic, Metabolic, and Immune Biomarkers in Blood and Urine for Early Colorectal Cancer Detection: A Multimarker Panel
Source: Diagnostics (Basel). 2026 Jun 6;16(12):1753. doi: 10.3390/diagnostics16121753 (PMC13298955; doi:10.3390/diagnostics16121753)
Supplement: Supplementary file 1 [file diagnostics-16-01753-s001.zip › Supplementary_ Table_S11.pdf]

Table S11. Subgroup-specific diagnostic performance of optimized multimarker panels (D1, D3, and D4) for colorectal cancer.

Models	Variables	Subgroup	**AUC**	**Sensitivity**	**Specificity**	**95%CI**	DeLong's p- values
**Model D4;** (mSEPT9 + DiAcSpm + NLR + PLR + LMR)	Age	<60	0.981	0.939	0.942	0.966–0.996	0.002*
		≥60	0.919	0.881	0.855	0.883–0.955	
	Tumor Volume(cm^3)	<5.88	0.893	0.796	0.887	0.837–0.944	0.003*
		≥5.88	0.979	0.935	0.933	0.968–0.99	
	Tumor Stage	Early Stage(I+II)	0.905	0.827	0.854	0.864–0.945	<0.001**
		Advanced Stage(III+IV)	0.994	1.000	0.967	0.991–1.000	
	N Stage	N0	0.907	0.763	0.925	0.869-0.945	(N0, N1) vs N2 <0.001**
		N1	0.994	0.975	0.979	0.989–1.000	
		N2	0.994	1.000	0.971	0.988-0.999	N0 vs N2 <0.857
	M stage	M0	0.939	0.837	0.929	0.913–0.965	<0.001**
		M1	0.998	1.000	0.988	0.994–1.000	
	Lymph node Invasion	Absent	0.913	0.782	0.925	0.876–0.950	<0.001**
		Present	0.989	0.969	0.942	0.982–0.996	
**Model D3;** (mSEPT9 + DiAcSpm + NLR + PLR + CEA + CA199 + CA125 + AFP)	Age	<60	0.960	0.880	0.958	0.946–0.994	<0.154
		≥60	0.942	0.899	0.917	0.914–0.971	
	Tumor Volume(cm$^{3)}$	<5.88	0.895	0.755	0.912	0.840–0.949	0.003*
		≥5.88	0.980	0.946	0.946	0.966–0.994	
	Tumor Stage	Early Stage(I+II)	0.908	0.827	0.900	0.867–0.949	<0.001**
		Advanced Stage(III+IV)	0.996	1.000	0.971	0.991–1.000	
	N stage	N0	0.912	0.892	0.904	0.872–0.953	<0.001**
		N1	0.993	0.975	0.958	0.986–1.000	<0.001**
		N2	0.994	1.000	0.971	0.988-1.000	<0.735
	M stage	M0	0.942	0.894	0.904	0.916–0.969	<0.001**
		M1	0.999	1.000	0.996	0.997–1.000	
	Lymph node Invasion	Absent	0.918	0..846	0.904	0.879–0.997	<0.001**
		Present	0.988	0.953	0.963	0.979–0.997	

**Model D1**; (mSEPT9 + DiAcSpm + NLR + PLR + LMR + CEA + CA199)	Age	<60	0.976	0.970	0.883	0.959–0.993	0.047*
		≥60	0.943	0.881	0.917	0.915–0.970	
	Tumor Volume(cm^{3})	<5.88	0.896	0.826	0.887	0.842–0.949	0.002*
		≥5.88	0.892	0.925	0.942	0.972–0.993	
	Tumor stage	Early Stage (I+II)	0.910	0.880	0.846	0.870–0.949	<0.001**
		Advanced Stage(III+IV)	0.996	1.000	0.975	0.991–1.000	
	N stage	N0	0.914	0.882	0.850	0.876–0.953	<0.001**
		N1	0.995	0.975	0.983	0.990–1.000	<0.001**
		N2	0.994	1.000	0.975	0.988–1.000	< 0.837
	M stage	M0	0.943	0.854	0.921	0.917–0.969	<0.001**
		M1	0.998	1.000	0.988	0.995–1.000	
	Lymph node Invasion	Absent	0.920	0.821	0.917	0.882–0.957	<0.001**
		Present	0.990	0.953	0.975	0.981–0.998	

Table S11.Performance of three optimized biomarker panels (D1, D3, and D4) stratified by clinicopathological variables: age (<60 vs. ≥60 years), tumor volume (<5.88 vs. ≥5.88 cm³), tumor stage (early: I+II vs. advanced: III+IV), N stage (N0, N1, N2), M stage (M0 vs. M1), and lymph node invasion (absent vs. present).

Models:

- D1: mSEPT9 + DiAcSpm + NLR + PLR + LMR + CEA + CA19-9

- D3:mSEPT9 + DiAcSpm + NLR + PLR + CEA + CA19-9 + CA125 + AFP

- D4:mSEPT9 + DiAcSpm + NLR + PLR + LMR

Metrics reported:

- AUC: Area under the ROC curve.

- Sensitivity (%) and Specificity (%): At the Youden-optimized threshold for each model.

- 95% CI: confidence interval for the AUC.

- DeLong’s p-values: for comparisons between subgroups (e.g., <60 vs. ≥60) within each model.

Comparator group: For all ROC analyses, the non-CRC group consisted of both colorectal polyp patients (n = 62) and non-malignant controls (hernia and hemorrhoid patients, n = 178); total non-CRC = 240.

Abbreviations: CRC, colorectal cancer; mSEPT9, methylated septin 9; DiAcSpm, N^1,N^{12}-diacetylspermine; NLR, neutrophil-to-lymphocyte ratio; PLR, platelet-to-lymphocyte ratio; LMR, lymphocyte-to-monocyte ratio; CEA, carcinoembryonic antigen; CA19-9, carbohydrate antigen 19-9; CA125, carbohydrate antigen 125; AFP, alpha-fetoprotein; AUC, area under the curve; CI, confidence interval.

Interpretation: The D4 panel consistently maintained high accuracy across all subgroups, demonstrating robustness for detecting colorectal cancer in diverse clinical settings. It performed particularly well in advanced stages (AUC = 0.994) and metastatic disease (AUC = 0.998), while also showing good performance in early-stage disease (AUC = 0.905). Significance levels:** *p < 0.05; **p < 0.001.
