# Supplementary material for: Hypoxia Associated Integration of Epigenetic, Metabolic, and Immune Biomarkers in Blood and Urine for Early Colorectal Cancer Detection: A Multimarker Panel
Source: Diagnostics (Basel). 2026 Jun 6;16(12):1753. doi: 10.3390/diagnostics16121753 (PMC13298955; doi:10.3390/diagnostics16121753)
Supplement: Supplementary file 1 [file diagnostics-16-01753-s001.zip › Supplementary_ Table_S2.pdf]

Table S2. Clinicopathological features between urinary N^1,N^{12}-diacetylspermine (DiAcSpm) positive(≥32.32 ng/mL) and negative(≥32.32 ng/mL) colorectal cancer patients.

Parameters	Overall	DiAcSpm-positive≥32.32 ng/mL	DiAcSpm-positive≥32.32 ng/mL	P-Value
Number	382	147(38.5)	235(61.5)	
Sex				0.481
Male	203	82(40.4)	121(59.6)	
Female	179	65(36.9)	114(63.1)	
Age				<0.001**
<60	204	57(27.9)	147(72.1)	
≥60	178	91(51.1)	87(48.9)	
Tumor Volume				
<5.88	49	31(63.3)	18(36.7)	0.072
≥5.88	93	72(77.4)	21(22.6)	
Location				0.482
Right Colon	38	30 (78.9)	8(21.1)	
Left Colon	65	47 (72.3)	18 (27.7)	
Rectum	39	26 (66.7)	13 (33.3)	
Gross Type				0.307
Ulcerative	80	60 (75.0)	20 (25.0)	
Polypoid	40	30 (75.0)	10 (25.0)	
Unknown	22	13 (59.1)	9 (40.9)	
Differentiation				0.238
Low	11	9 (81.8)	2 (18.2)	
Low-Moderate	55	37 67.3)	18 (34.7)	
Moderate	52	36 (69.2)	16 (30.8)	
High	24	21 (87.5)	3 (12.5)	
Tumor stage				0.018*
Stage I	35	21 (60%)	14 (40%)	
Stage II	40	25 (62.5%)	15 (37.5%)	
Stage III	48	41 (85.4%)	7 (14.6%)	
Stage IV	19	16 (84.2%)	3 (15.8%)	
pT stage				0.040*
T1	6	6 (100%)	0 (0.0%)	
T2	29	17 (58.6%)	12 (41.4%)	
T3	77	54 (70.1%)	23 (29.9%)	
T4	30	26 (86.7%)	4 (13.3%)	
N stage				0.069
N0	76	49 (64.5%)	27 (35.5%)	
N1	40	33 (17.5%)	7 (82.5%)	
N2	26	21 (80.8%)	5 (19.2%)	
M stage				0.221
M0	123	87 (70.7)	36 (29.3%)	
M1	19	16 (84.2%)	3 (16.8%)	

Lymph node invasion				0.330
Absent	78	54(69.2)	24(30.8)	
Present	64	49(76.6)	15(23.4)	
Vascular invasion				0.932
Absent	70	51(72.9)	19(27.1)	
Present	72	52(72.2)	20(27.8)	
Perineural invasion				0.493
Absent	83	62(74.7)	21(25.3)	
Present	59	41(69.5)	18(30.5)	

Table S2. Comparison of clinicopathological features between DiAcSpm-positive (≥32.32 ng/mL) and DiAcSpm-negative (<32.32 ng/mL) colorectal cancer patients (n = 382). Urinary DiAcSpm concentrations are reported as absolute values (ng/mL) without creatinine normalization; the threshold (≥32.32 ng/mL) was determined by ROC analysis (see Section 2.7). Values are presented as frequencies with percentages in parentheses. Statistical significance was assessed using the χ^2 test or Fisher's exact test, as appropriate.

Abbreviations: DiAcSpm, N^1,N^{12}-diacetylspermine; T stage, tumor invasion depth; TNM, tumor-node-metastasis.

Interpretation: DiAcSpm positivity was significantly associated with advancing T stage and overall TNM stage ($p < 0.05$ for both) but was not associated with nodal status (N stage) or distant metastasis (M stage). No significant associations were observed with sex, tumor location, gross tumor type, tumor differentiation, vascular invasion, or perineural invasion.

Note: The lack of creatinine normalization means that absolute DiAcSpm values may be influenced by urine dilution; this limitation is discussed in the main text (Section 4, Discussion).

Significance levels: *$p < 0.05$; **$p < 0.01$.
