# Supplementary material for: Hypoxia Associated Integration of Epigenetic, Metabolic, and Immune Biomarkers in Blood and Urine for Early Colorectal Cancer Detection: A Multimarker Panel
Source: Diagnostics (Basel). 2026 Jun 6;16(12):1753. doi: 10.3390/diagnostics16121753 (PMC13298955; doi:10.3390/diagnostics16121753)
Supplement: Supplementary file 1 [file diagnostics-16-01753-s001.zip › Supplementary_ Table_S3.pdf]

Table S3: Comparative distributions of epigenetic, metabolic, inflammatory, and classical serum biomarkers across diagnostic groups (CRC, polyps, non-cancerous controls).

Biomarker Category	Biomarker	CRC (n=142)	Polyps (n=62)	Non-Cancerous Controls (n=178)	CRC vs. Polyps	CRC vs. Controls	Polyps vs. Controls
**Epigenetic marker**	mSEPT9 (%)	15.2 (8.9–25.1)	8.0 (5.6–11.3)	5.3 (2.2–7.2)	<0.001**	<0.001**	<0.001**
**Metabolite marker**	DiAcSpm (ng/mL)	33.8 (31.4–35.4)	30.0 (28.7–32.8)	29.2 (27.7–31.2)	<0.001**	<0.001**	<0.007*
**Classic Tumor Markers**	CEA (ng/mL)	3.8 (2.1–8.5)	2.6 (1.9–3.5)	2.7 (1.9–3.4)	<0.001**	<0.001**	0.860
	CA125 (U/mL)	9.8 (6.8–20.9)	10.1 (7.9–12.7)	15.6 (10.3–20.2)	0.0483*	0.001*	<0.001**
	CA199 (U/mL)	17.1 (8.2–39.7)	13.4 (9.8–17.8)	10.5 (6.4–14.7)	0.067	<0.001**	0.044*
	AFP (ng/mL)	3.3 (2.4–4.8)	2.7 (2.2–3.4)	3.5 (2.3–4.8)	0.003*	0.849	0.001*
**Inflammatory Indices**	NLR (Ratio)	3.3 (2.5–4.1)	2.2 (1.7–2.6)	2.1 (1.8–2.6)	<0.001**	<0.001**	0.486
	LMR (Ratio)	2.5 (1.8–3.2)	3.4 (2.7–4.4)	4.1 (2.8–5.3)	<0.001**	<0.001**	0.187
	PLR (Ratio)	194 (151–250)	124 (111–196)	122 (106–150)	<0.001**	<0.001**	0.043*

Table S3. Comparative distributions of epigenetic (mSEPT9), metabolic (DiAcSpm), inflammatory (NLR, PLR, LMR), and classical serum biomarkers (CEA, CA19-9, CA125, AFP) across diagnostic groups: colorectal cancer (CRC, n = 142), colorectal polyps (n = 62), and non-cancer controls (n = 178). Values are presented as median (interquartile range). Non-parametric comparisons were performed using the Kruskal–Wallis test followed by Dunn's post hoc correction for pairwise comparisons (CRC vs. polyps, CRC vs. controls, polyps vs. controls).

Abbreviations: CRC, colorectal cancer; mSEPT9, methylated septin 9 (%); DiAcSpm, N^1,N^{12}-diacetylspermine (ng/mL); CEA, carcinoembryonic antigen (ng/mL); CA125, carbohydrate antigen 125 (U/mL); CA19-9, carbohydrate antigen 19-9 (U/mL); AFP, alpha-fetoprotein (ng/mL); NLR, neutrophil-to-lymphocyte ratio; PLR, platelet-to-lymphocyte ratio; LMR, lymphocyte-to-monocyte ratio.

Interpretation: CRC patients showed significantly higher levels of mSEPT9, DiAcSpm, NLR, PLR, CEA, CA19-9, and CA125, and significantly lower LMR, compared to polyps and controls ($p < 0.05$ for all). Polyp patients had intermediate values between CRC and controls for most biomarkers. Pairwise comparisons that were not statistically significant are indicated by the absence of an asterisk or by a p-value > 0.05 (e.g., CEA, polyps vs. controls: p = 0.860).

Significance levels: *p < 0.05; **p < 0.01; ***p < 0.001. Exact p-values are shown in the table.
