# Supplementary material for: Hypoxia Associated Integration of Epigenetic, Metabolic, and Immune Biomarkers in Blood and Urine for Early Colorectal Cancer Detection: A Multimarker Panel
Source: Diagnostics (Basel). 2026 Jun 6;16(12):1753. doi: 10.3390/diagnostics16121753 (PMC13298955; doi:10.3390/diagnostics16121753)
Supplement: Supplementary file 1 [file diagnostics-16-01753-s001.zip › Supplementary_ Table_S4.pdf]

Table S4. Associations of classical serum tumor markers and systemic inflammatory indices with clinicopathological characteristics in colorectal cancer patients.

Parameter	CEA Median, IQR	P	CA125 Median, IQR	P	CA199 Median, IQR	P	AFP Median, IQR	P	NLR Median, IQR	P	LMR Median, IQR	P	PLR Median,IQR	P
Sex		0.224		0.292		0.664		0.835		0.634		0.040*		0.527
Male	3.53[2.12-8.25]		12.53[8.09-27.68]		22.52[9.28-46.87]		3.25[2.40-4.76]		3.46[2.54–4.17]		2.39 1.80-3.30		207.36[149.38-250.47]	
Female	4.49[2.09–9.47]		8.42(6.25-17.28)		13.90(7.58-69.31		3.26[2.39-5-34)		3.20[2.39-4.04]		2.47[1.78-3.11]		190.83[150.47-253.26	
Age		0.047*		0.287		<0.001**		0.374		<0.001**		<0.001**		<0.001**
<60	4.20[2.29-6.47]		9.45[7.20-19.75]		13.10[6.91-29.65]		3.38[2.42-5.83]		3.20[2.46-3.60]		2.50[1.79-3.22]		211.27[152.23-256.88]	
≥60	3.75[2.09-9.37]		9.80-[6.57-21.60]		19.90[8.88-43.87]		3.18[2.41-4.53]		3.33[2.44-4.21]		2.45[1.79-3.19]		191.30[149.88-249.42]	
Stage		0.202		0.352		0.141		0.659		<0.001**		0.154		0.090
I	4.56[2.34–8.87]		14.60[7.36-19.30]		23.50 [8.18–64.86]		3.38 [2.25–5.13]		2.83[2.33–3.51]		1.92[1.61-2.46]		1.77.23[148.89-250.79]	
II	5.07[2.38-14.20]		9.22[5.16-20.93]		10.14 [6.91–29.83]		3.51 [2.41–5.63]		2.88[2.30–3.6]		1.98[1.54-2.63]		188.03[123.96-215.43]	
III	2.79[1.76-6.78]		9.60[8.01-27.72]		19.85 [9.28–37.89]		3.25 [2.47–4.66]		3.54[2.68–4.52]		1.68[1.38-2.34]		211.19[158.46-263.39]	
IV	3.34[1.64-8.25]		7.63[6.48-17.00]		24.50 [9.15–54.03]		2.90 [2.41–3.99]		4.63[3.56–5.38]		1.63[1.22-2.69]		218.03[169.31-286.81]	
location		0.531		0.796		0.086		0.039*		0.098		0.051		0.586
Right Colon	3.08[1.80-9.80]		12.12[7.21–25.73]		27.00 [9.15–55.97]		2.83 [2.27–3.74]		3.52[2.87–4.78]		2.08[1.53–2.87]		188.73 [153.46–244.18]	
Left Colon	3.75[2.08-6.98]		9.77 [6.48–20.77]		11.09[8.10–28.90]		3.70 [2.52–5.58]		3.07[2.46–3.94]		2.49[1.95–3.26]		209.17 [161.19–258.24]	
Rectum	4.20[2.30-12.00]		9.38[6.48–21.60]		24.28[8.01-69.09]		3.18[2.41–4.69]		3.20 2.34–3.89		2.65(2.15–-3.27)		175.17 [131.22–246.39]	

T stage		0.211		0.819		0.648		0.293		0.015*		0.818		0.959
T1	20.74[4.84–62.13]		13.69 [7.30–17.97]		31.03[6.63–94.28]		3.49 [2.12–4.63]		2.99 [2.41–3.38]		2.50 [1.78 – 3.18]		179.60[120.68–243.87]	
T2	3.65 (2.28–7.47		14.60 [7.20–21.60]		23.36[8.31–53.71]		3.18[2.06–5.32]		2.77 [1.80–3.54]		2.40[1.79–3.37]		182.09 [149.91–265.20]	
T3	3.87[2.21–8.25]		9.46[6.71–19.41]		19.90[9.05–34.62]		3.49 [2.46–5.41]		3.29[2.51–4.21]		2.45 [1.75–3.15]		197.52 [147.77–247.62]	
T4	2.74[1.50–9.13]		8.08[6.62–24.41]		9.61[6.94–33.10]		2.81[2.37–3.81]		3.53[2.89–5.24]		2.76[1.87 –3.32]		204.19 [156.35–251.04]	
N stage		0.488		0.090		0.219		0.812		0.001*		0.033*		0.002*
N0	4.56[2.22–8.97]		10.44 [6.30–19.09]		11.57 [7.46–37.46]		3.49 [2.43–5.21]		2.88 [2.30–3.69]		2.65[1.98–3.35]		178.06 [136.74–224.19]	
N1	3.15[2.12–6.32]		11.15 [8.39–28.13]		18.45 [8.70–51.72]		3.04 [2.30–4.66]		3.60 [3.15–5.11]		2.19 [1.68 – 2.73]		236.49 [174.58–284.29]	
N2	3.20 [1.65–8.25]		8.62 [6.38–15.13]		25.54[10.55–37.08]		3.11 [2.41–4.13]		3.56 [2.51–4.87]		2.31 [1.51 –3.18]		186.70 [150.23–222.43]	
M stage		0.617		0.459		0.569		0.223		0.007*		0.210		0.136
M0	3.87 [2.21–8.87]		10.20 [6.94–21.60]		17.10 [8.07–38.78]		3.33 [2.41–5.23]		3.20 [2.40–3.88]		2.46 [1.92-3.20]		191.30 [148.33–246.39]	
M1	3.34 [1.64–8.25]		7.63 [6.48–17.00]		24.50 [9.15–54.03]		2.90 [2.41–3.99]		4.25 [2.95–5.63]		2.29 [1.63-3.11]		218.03 [169.31–286.81]	
Cancer diff		0.089		0.879		0.470		0.110		0.123		0.143		0.009*
Low	2.74 [1.50–13.60]		10.68 [3.57–19.10]		38.60 [8.37–73.58]		4.47[2.90–5.84]		2.58[2.16-3.64]		3.10 [2.33–3.57]		175.17 [100.00–197.26]	
Low-Moderate	5.21 [2.54–14.78]		10.80 [6.30–24.15]		20.10 [8.01–51.60]		3.14 [2.22–4.69]		3.13[2.48-3.10]		2.52 [1.69–3.31]		182.09 [127.52–236.80]	
Moderate	3.61 [2.13–6.42]		9.46 [7.06–19.20]		13.09 [8.29–30.25]		3.66 [2.59–5.44]		3.37[2.38-3.87]		2.46 [1.96–3.19]		200.52 [162.66–254.57]	
High	2.72 [1.54–7.91]		9.68 [6.87–28.75]		22.04 [9.46–28.99]		2.88 [2.46–3.82]		3.92[2.54-5.42]		1.95 [1.56–3.02]		239.21 [175.96–290.65]	

Gross type		0.694		0.027 *		0.840		0.666		0.742		0.897		0.766
Ulcerative	3.75 [2.14–8.80]		8.62 [6.38–18.04]		14.83 [8.01-42.53]		3.01[2.41–4.79]		3.20 (2.48–4.24)		2.45 [1.92-3.19]		180.00 [153.45–248.72]	
Polypoid	3.44 [2.13–7.66]		9.76 (7.47–37.41)		19.85 [9.18–34.40]		3.56 [2.45–4.69]		3.22 [2.28–4.13]		2.44[1.73-3.15]		205.07 [148.47–262.85]	
Unknown	4.68 [1.94–30.60]		16.85 (10.45–25.56)		16.85 [7.47-37.41]		3.86 [2.24–5.82]		3.49 [2.56–3.78]		2.54[1.60-3.50]		207.34 [160.95–238.96]	
Vascular Invasion		0.811		0.213		0.023*		0.423		0.043*		0.736		0.519
Absent	3.54[2.23-7.19]		11.65[7.38-33.65]		11.55[7.38-33.65]		3.17[2.24-4.50]		3.10[2.34-3.74]		2.44[1.69-3.31]		197.07[153.46-251.21]	
Present	4.10[1.64-9.68]		8.70[6.69-18.77]		26.80[9.18-42.41]		3.41[2.49-5.09]		3.44[2.55-4.63]		2.46[1.93-3.17]		190-83[148.47-253.36]	

Table S4. Associations of classical serum tumor markers (CEA, CA19-9, CA125, AFP) and systemic inflammatory indices (NLR, PLR, LMR) with clinicopathological characteristics in colorectal cancer patients (n = 142). Data are presented as median (interquartile range, IQR). Comparisons across clinicopathological variables – including sex, age (<60 vs. ≥60 years), tumor stage (I–IV), tumor location (right colon, left colon, rectum), T stage (T1–T4), N stage (N0–N2), M stage (M0/M1), histological differentiation (low, low–moderate, moderate, high), gross tumor type (ulcerative, polypoid, unknown), and vascular invasion (absent/present) – were performed using non-parametric tests. The Mann–Whitney U test was used for two-group comparisons, and the Kruskal–Wallis test was applied for three or more groups, with significance set at $p < 0.05$.

Abbreviations: CEA, carcinoembryonic antigen; CA125, carbohydrate antigen 125; CA19-9, carbohydrate antigen 19-9; AFP, alpha-fetoprotein; NLR, neutrophil-to-lymphocyte ratio; LMR, lymphocyte-to-monocyte ratio; PLR, platelet-to-lymphocyte ratio; IQR, interquartile range.

Interpretation: Inflammatory indices, particularly NLR and PLR, showed significant variation with tumor burden and nodal status, supporting their potential role as adjunctive biomarkers alongside classical serum markers. Classical serum markers generally demonstrated weaker and less consistent associations with clinicopathological parameters.

Significance levels: *$p < 0.05$; **$p < 0.01$. Exact p-values are shown in the table.
