# Supplementary material for: Hypoxia Associated Integration of Epigenetic, Metabolic, and Immune Biomarkers in Blood and Urine for Early Colorectal Cancer Detection: A Multimarker Panel
Source: Diagnostics (Basel). 2026 Jun 6;16(12):1753. doi: 10.3390/diagnostics16121753 (PMC13298955; doi:10.3390/diagnostics16121753)
Supplement: Supplementary file 1 [file diagnostics-16-01753-s001.zip › Supplementary_ Table_S5.pdf]

Table S5. Pairwise comparison of diagnostic performance between epigenetic (mSEPT9), metabolic (DiAcSpm), and classical serum biomarkers for colorectal cancer detection using McNemar's test.

**Biomarker 1**	**Biomarker 2**	**n10**	**n01**	**OR**	**95% CI**	**p-value (exact)**	**p-value (FDR)**	**Interpretation**
mSEPT9	DiAcSpm	47	61	0.77	0.53 – 1.13	0.211	0.226	No difference
mSEPT9	CEA	92	23	4.00	2.53 – 6.32	5.8×10^{-11}	1.1×10^{-10}	Favor mSEPT9
mSEPT9	CA125	110	24	4.58	2.95 – 7.13	2.4×10^{-14}	5.9×10^{-14}	Favor mSEPT9
mSEPT9	CA19-9	101	20	5.05	3.13 – 8.16	3.3×10^{-14}	7.1×10^{-14}	Favor mSEPT9
mSEPT9	AFP	122	6	20.33	8.96 – 46.15	3.3×10^{-29}	2.5×10^{-28}	Strongly favor mSEPT9
DiAcSpm	CEA	99	16	6.19	3.65 – 10.49	8.6×10^{-16}	2.6×10^{-15}	Favor DiAcSpm
DiAcSpm	CA125	125	25	5.00	3.25 – 7.68	3.4×10^{-17}	1.3×10^{-16}	Favor DiAcSpm
DiAcSpm	CA19-9	111	16	6.94	4.11 – 11.72	1.1×10^{-18}	5.6×10^{-18}	Favor DiAcSpm
DiAcSpm	AFP	138	8	17.25	8.46 – 35.18	1.0×10^{-31}	1.5×10^{-30}	Strongly favor DiAcSpm
CEA	CA125	52	35	1.49	0.97 – 2.28	0.086	0.107	No difference
CEA	CA19-9	35	23	1.52	0.90 – 2.58	0.148	0.171	No difference
CEA	AFP	64	17	3.76	2.21 – 6.43	1.4×10^{-7}	2.4×10^{-7}	Favor CEA
CA125	CA19-9	36	41	0.88	0.56 – 1.37	0.649	0.649	No difference
CA125	AFP	45	15	3.00	1.67 – 5.38	1.4×10^{-4}	1.8×10^{-4}	Favor CA125
CA19-9	AFP	49	14	3.50	1.93 – 6.34	1.1×10^{-5}	1.7×10^{-5}	Favor CA19-9

Table S5. Pairwise McNemar's test comparing plasma methylated SEPT9 (mSEPT9), urinary N^1,N^{12}-diacetylspermine (DiAcSpm), and classical serum markers (CEA, CA19-9, CA125, AFP) in the same cohort of 382 participants. The non-CRC comparator group consisted of both colorectal polyp patients (n = 62) and non-malignant controls (hernia and hemorrhoid patients, n = 178), totaling 240 non-CRC individuals.

Column definitions: n10: number of patients positive for Biomarker 1 but negative for Biomarker 2.

-n01: number of patients positive for Biomarker 2 but negative for Biomarker 1.

- OR (odds ratio): (n10 / n01). An OR > 1 indicates that Biomarker 1 is more frequently positive than Biomarker 2.

- 95% CI: confidence interval of the odds ratio.

- p-value (exact): two-sided exact McNemar's p-value.

- p-value (FDR):Benjamini–Hochberg false discovery rate adjusted p-value for multiple comparisons.

Interpretation: mSEPT9 and DiAcSpm consistently outperformed all classical serum markers (OR range for mSEPT9: 4.00–20.33; for DiAcSpm: 5.00–17.25; all FDR-adjusted $p < 0.001$). No significant differences in detection performance were observed between mSEPT9 and DiAcSpm (OR = 0.77, 95% CI: 0.53–1.13, FDR-adjusted $p = 0.226$), nor among most classical serum marker pairs.

Significance levels: FDR-adjusted $p < 0.05$ was considered statistically significant.
