# Supplementary material for: Hypoxia Associated Integration of Epigenetic, Metabolic, and Immune Biomarkers in Blood and Urine for Early Colorectal Cancer Detection: A Multimarker Panel
Source: Diagnostics (Basel). 2026 Jun 6;16(12):1753. doi: 10.3390/diagnostics16121753 (PMC13298955; doi:10.3390/diagnostics16121753)
Supplement: Supplementary file 1 [file diagnostics-16-01753-s001.zip › Supplementary_ Table_S6.pdf]

Table S6. Diagnostic accuracy of individual non-invasive biomarkers for colorectal cancer detection

**Biomarker**	**AUC[95% CI]**	**Sensitivity%**	**Specificity%**	**p-value**
AFP	0.543 (0.482 – 0.603)	21.8	86.7	0.165
CA125	0.558 (0.493 – 0.623)	45.1	78.3	0.060
CA199	0.649 (0.587 – 0.712)	45.1	94.6	<0.001**
CEA	0.660 (0.597 – 0.723)	43.7	96.3	<0.001**
LMR	0.746 (0.697 – 0.796)	67.6	73.3	<0.001**
NLR	0.780 (0.730 – 0.829)	66.9	80.0	<0.001**
PLR	0.781 (0.731 – 0.831)	65.5	82.1	<0.001**
DiAcSpm	0.831 (0.788 – 0.874)	72.5	82.9	<0.001**
mSEPT9	0.843 (0.800 – 0.886)	73.2	87.5	<0.001**

Table S6: Receiver operating characteristic (ROC) analysis comparing plasma epigenetic (mSEPT9), urinary metabolic (N^1,N^{12}-diacetylspermine, DiAcSpm), systemic inflammatory (NLR, PLR, LMR), and classical serum (CEA, CA19-9, CA125, AFP) biomarkers for distinguishing colorectal cancer (CRC) from non-CRC individuals. The non-CRC comparator group consisted of both colorectal polyp patients (n = 62) and non-malignant controls (hernia and hemorrhoid patients, n = 178), totaling 240 non-CRC participants.

Metrics reported: Area under the ROC curve (AUC) with 95% confidence interval (CI), sensitivity (%), specificity (%), and p-value (testing whether the AUC is significantly different from 0.5, i.e., no discrimination).

Abbreviations: CRC, colorectal cancer; mSEPT9, methylated septin 9; DiAcSpm, N^1,N^{12}-diacetylspermine; NLR, neutrophil-to-lymphocyte ratio; PLR, platelet-to-lymphocyte ratio; LMR, lymphocyte-to-monocyte ratio; CEA, carcinoembryonic antigen; CA19-9, carbohydrate antigen 19-9; CA125, carbohydrate antigen 125; AFP, alpha-fetoprotein.

Interpretation: Epigenetic (mSEPT9) and urinary metabolic (DiAcSpm) biomarkers demonstrated the highest discriminatory performance (AUC 0.843 and 0.831, respectively), substantially outperforming classical serum markers (CEA, AUC 0.660; CA19-9, AUC 0.649) and systemic inflammatory indices (AUC range: 0.746–0.781). All reported biomarkers showed statistically significant discrimination compared to random chance (AUC = 0.5).

Significance levels: $^{**}p < 0.01$.
