# Supplementary material for: Hypoxia Associated Integration of Epigenetic, Metabolic, and Immune Biomarkers in Blood and Urine for Early Colorectal Cancer Detection: A Multimarker Panel
Source: Diagnostics (Basel). 2026 Jun 6;16(12):1753. doi: 10.3390/diagnostics16121753 (PMC13298955; doi:10.3390/diagnostics16121753)
Supplement: Supplementary file 1 [file diagnostics-16-01753-s001.zip › Supplementary_ Table_S7.pdf]

Table S7: Diagnostic performance of multimarker models for colorectal cancer detection.

Model Group	Biomarker Combination	AUC (95% CI)	Sensitivity (%)	Specificity (%)	p-value
**A. Epigenetic/ Metabolic + Inflammatory Models**					
**A1**	mSEPT9 + NLR	0.895 (0.864–0.927)	83.8	80.8	<0.001**
**A2**	DiAcSpm + NLR	0.896 (0.862–0.930)	86.6	79.6	<0.001**
**A3**	mSEPT9 + NLR + PLR	0.916 (0.888–0.945)	84.5	84.6	<0.001**
**A4**	mSEPT9 + DiAcSpm	0.896 (0.861–0.931)	77.5	91.7	<0.001**
**A5**	mSEPT9 + DiAcSpm + PLR	0.918 (0.888–0.948)	80.3	90.0	<0.001**
**A6**	mSEPT9 + DiAcSpm + PLR + NLR	0.940 (0.918–0.967)	85.2	92.5	<0.001**
**B. Inflammatory + Classical Marker Models**					
**B1**	NLR + PLR + CEA + CA19-9	0.879 (0.844–0.915)	80.3	79.6	<0.001**
**B2**	NLR + CEA + CA19-9	0.818 (0.773–0.863)	80.3	74.2	<0.001**
**B3**	PLR + CEA + CA19-9	0.832 (0.787–0.876)	79.6	76.7	<0.001**
**B4**	PLR + CA19-9	0.812 (0.765–0.860)	78.2	77.1	<0.001**
**B5**	NLR + PLR + LMR	0.888 (0.854–0.922)	87.3	73.3	<0.001**
**B6**	NLR + PLR + CEA	0.877 (0.842–0.912)	78.9	80.0	<0.001**
**B7**	NLR + PLR + CA19-9	0.868 (0.831–0.905)	81.0	76.7	<0.001**
**B8**	NLR + PLR + LMR + CEA + CA19-9	0.899 (0.867–0.932)	93.2	80.3	<0.001**
**B9**	NLR + PLR + LMR + CA19-9	0.894 (0.860–0.927)	77.5	85.8	<0.001**
**C. Classical + Epigenetic/Metabolic Models**					
**C1**	CEA + CA19-9	0.722 (0.664–0.781)	54.2	89.6	<0.001**
**C2**	CEA + CA19-9 + DiAcSpm	0.852 (0.813–0.892)	75.4	83.8	<0.001**
**C3**	CEA + CA19-9 + mSEPT9	0.888 (0.852–0.924)	84.5	84.2	<0.001**
**C4**	CEA + CA19-9 + mSEPT9 + DiAcSpm + LMR	0.927 (0.899–0.955)	88.0	87.1	<0.001**
**C5**	CEA + CA19-9 + mSEPT9 + DiAcSpm	0.914 (0.883–0.946)	83.1	90.0	<0.001**
**C6**	CEA + mSEPT9 + DiAcSpm	0.903 (0.869–0.937)	83.1	90.8	<0.001**
C7	CEA + CA19-9 + mSEPT9 + DiAcSpm + NLR	0.932 (0.904–0.959)	85.2	91.7	<0.001**

**C8**	CEA + CA19-9 + mSEPT9 + DiAcSpm + PLR	0.930 (0.903–0.957)	83.8	89.2	<0.001**
**D. Full Multimarker Models**					
**D1**	mSEPT9 + DiAcSpm + NLR + PLR + LMR + CEA + CA19-9	0.950 (0.928–0.973)	86.6	93.5	<0.001**
**D2**	mSEPT9 + DiAcSpm + CEA + CA19-9 + CA125 + AFP	0.918 (0.887–0.948)	82.4	90.4	<0.001**
**D3**	mSEPT9 + DiAcSpm + NLR + PLR + CEA + CA19-9 + CA125 + AFP	0.950 (0.927–0.973)	90.8	90.4	<0.001**
**D4**	mSEPT9 + DiAcSpm + NLR + PLR + LMR	0.947 (0.924–0.970)	85.9	92.9	<0.001**
**D5**	NLR + PLR + LMR + CEA + CA19-9	0.899 (0.867–0.932)	80.3	83.8	<0.001**
**D6**	mSEPT9 + NLR + PLR + CEA + CA19-9 + CA125 + AFP	0.926 (0.900–0.953)	79.6	92.9	<0.001**

Table S7: Evaluation of multimarker combinations integrating plasma epigenetic (mSEPT9), urinary metabolic (N^1,N^{12}-diacetylspermine, DiAcSpm), systemic inflammatory (NLR, PLR, LMR), and classical serum (CEA, CA19-9, CA125, AFP) biomarkers for distinguishing colorectal cancer (CRC) from non-CRC individuals. The non-CRC comparator group consisted of both colorectal polyp patients (n = 62) and non-malignant controls (hernia and hemorrhoid patients, n = 178), totaling 240 non-CRC participants.

Metrics reported: Area under the ROC curve (AUC) with 95% confidence interval (CI), sensitivity (%), specificity (%), and p-value (from DeLong test comparing the AUC to a null value of 0.5).

Model groups:

- Group A: Epigenetic/metabolic + inflammatory models.

- Group B: Inflammatory + classical marker models.

- Group C: Classical + epigenetic/metabolic models.

- Group D: Full multimarker models (combining all three biomarker classes).

Abbreviations: mSEPT9, methylated septin 9; DiAcSpm, N^1,N^{12}-diacetylspermine; NLR, neutrophil-to-lymphocyte ratio; PLR, platelet-to-lymphocyte ratio; LMR, lymphocyte-to-monocyte ratio; CEA, carcinoembryonic antigen; CA19-9, carbohydrate antigen 19-9; CA125, carbohydrate antigen 125; AFP, alpha-fetoprotein.

Interpretation: All models showed statistically significant discrimination ($p < 0.001$). The highest AUC values were observed in the full multimarker panels (Group D), with models D1, D3, and D4 achieving AUCs of 0.950, 0.950, and 0.947, respectively, indicating superior discriminatory power of integrative biomarker strategies over classical markers alone. The streamlined D4 model (mSEPT9 + DiAcSpm + NLR + PLR + LMR) achieved performance comparable to larger panels while using fewer biomarkers.

Significance levels: **$p < 0.001$ for all models.
