# Supplementary material for: Hypoxia Associated Integration of Epigenetic, Metabolic, and Immune Biomarkers in Blood and Urine for Early Colorectal Cancer Detection: A Multimarker Panel
Source: Diagnostics (Basel). 2026 Jun 6;16(12):1753. doi: 10.3390/diagnostics16121753 (PMC13298955; doi:10.3390/diagnostics16121753)
Supplement: Supplementary file 1 [file diagnostics-16-01753-s001.zip › Supplementary_ Table_S8.pdf]

Table S8. Univariate and multivariable logistic regression analysis of demographic factors and biomarkers associated with colorectal cancer risk.

**Binary Logistic Regression**	Biomarker / Variable	R^2	B	SE	Wald χ^2	P	OR	95% CI
Univariate binary logistic regression	**Demographic**							
	Sex	0.000	-0.024	0.212	0.013	0.909	0.976	0.644–1.479
	Age range (ref <50)							
	50–59	–	1.641	0.475	12.37	<0.001	5.16	2.07–12.87
	60–69	–	2.786	0.460	36.73	<0.001	16.22	6.59–39.9
	≥70	–	4.808	0.676	50.59	<0.001	122.4	32.5–461
	**Epigenetic/metabolic**							
	mSEPT9	0.315	0.209	0.0241	75.2	<0.001	1.233	1.179–1.296
	DiAcSpm	0.267	0.475	0.0504	88.8	<0.001	1.608	1.463–1.783
	**Inflammatory markers**							
	NLR	0.196	1.108	0.136	66.4	<0.001	3.027	2.32–3.95
	PLR	0.190	0.0189	0.00229	68.5	<0.001	1.019	1.015–1.024
	**LMR**	**0.136**	**-0.695**	**0.0976**	**50.7**	**<0.001**	**0.499**	**0.412–0.605**
	**Classical markers**							
	CEA	0.041	0.0496	0.0154	10.40	0.0013	1.051	1.024–1.089
	CA199	0.058	0.0185	0.00489	14.33	0.00015	1.019	1.010–1.030
	CA125	0.0046	0.0139	0.00913	2.31	0.128	1.014	0.996–1.032
	AFP	0.0125	0.0274	0.0195	1.96	0.161	1.028	0.989–1.067
Multivariate binary logistic regression	**Epigenetic/metabolic**							
		mSEPT9	0.137	0.030	21.38	<0.001	1.147	1.082–1.215
		DiAcSpm	0.394	0.074	28.61	<0.001	1.482	1.283–1.713
	**Inflammatory markers**							
		NLR	1.030	0.209	24.39	<0.001	2.800	1.861–4.214
		PLR	0.011	0.003	10.93	0.001	1.011	1.004–1.017
		**LMR**	**-0.435**	**0.156**	**7.79**	0.005	**0.648**	**0.477–0.879**
	**Classical markers**							
		CEA	0.010	0.010	1.04	0.309	1.010	0.991–1.030

		CA199	0.008	0.003	7.15	0.007	1.008	1.002–1.014
		CA125	0.009	0.019	0.21	0.644	1.009	0.972–1.047
		AFP	0.035	0.035	0.97	0.325	1.035	0.966–1.109

Table S8. Binary logistic regression models examining associations with colorectal cancer (CRC) status (1 = CRC, 0 = non-CRC). The non-CRC comparator group includes both colorectal polyp patients (n = 62) and non-malignant controls (hernia and hemorrhoid patients, n = 178), totaling 240 non-CRC participants. Complete case analysis was performed (N = 382).

Metrics reported:
- R^2: McFadden's pseudo R^2 (univariate models only).
- B: regression coefficient (log odds scale).
- SE: standard error.
- Wald χ^2: Wald chi-square statistic.
- p: p-value (two-tailed).
- OR: odds ratio.
- 95% CI: confidence interval for the odds ratio.

Categorical variables: Age range is categorical with <50 years as the reference category. Sex is binary (male/female).

Abbreviations: CRC, colorectal cancer; LMR, lymphocyte-to-monocyte ratio (positive values); a higher LMR is protective (OR < 1).

Multivariable model: Includes all biomarkers listed. McFadden's R^2 for the multivariable model is 0.603.

Interpretation: In univariate analysis, age ≥70 years, mSEPT9, DiAcSpm, NLR, PLR, LMR, and classical markers (CEA, CA19-9, CA125, AFP) were all significantly associated with CRC status. After multivariable adjustment, mSEPT9, DiAcSpm, NLR, PLR, and LMR remained independently associated with CRC, whereas most classical markers lost statistical significance, indicating that their predictive value was largely explained by overlap with stronger biological signals captured by the integrated biomarker domains.

Significance levels: ***p < 0.001; **p < 0.01; *p < 0.05.

Note: The final D4 model (excluding classical markers) is presented in Supplementary Table S17.
