# Supplementary material for: Hypoxia Associated Integration of Epigenetic, Metabolic, and Immune Biomarkers in Blood and Urine for Early Colorectal Cancer Detection: A Multimarker Panel
Source: Diagnostics (Basel). 2026 Jun 6;16(12):1753. doi: 10.3390/diagnostics16121753 (PMC13298955; doi:10.3390/diagnostics16121753)
Supplement: Supplementary file 1 [file diagnostics-16-01753-s001.zip › Supplementary_ Table_S9.pdf]

Table S9. Multivariable logistic regression odds ratios for colorectal cancer detection including all nine biomarkers simultaneously.

Biomarker	OR (95% CI)	Z-score (ORs per 1 SD increase)	p-value
mSEPT9	1.15 (1.08–1.22)	4.62	<0.001 ***
DiAcSpm	1.48 (1.28–1.71)	5.35	<0.001 ***
NLR	2.80 (1.86–4.21)	4.94	<0.001 ***
PLR	1.01 (1.00–1.02)	3.31	0.001 **
LMR	0.65 (0.48–0.88)	2.79	0.005 **
CEA	1.01 (0.99–1.03)	1.02	0.309

Table S9. Multivariable logistic regression model including all nine biomarkers simultaneously (mSEPT9, DiAcSpm, NLR, PLR, LMR, CEA, CA19-9, CA125, AFP) without adjustment for age or sex. Analysis was performed on 382 complete cases (non-CRC group includes colorectal polyps, n = 62, and non-malignant controls, n = 178; total non-CRC = 240).

Metrics reported:

- OR (95% CI): odds ratio with 95% confidence interval. ORs represent the change in the odds of colorectal cancer (CRC) per 1-unit increase in each biomarker on its original (raw) scale.

- Z-score: Wald statistic (coefficient divided by its standard error) for the standardized model (i.e., per one standard deviation increase in each biomarker). This allows direct comparison of effect magnitudes across biomarkers measured on different scales.

- p-value: two-tailed significance level.

Interpretation: LMR (lymphocyte-to-monocyte ratio) is defined as LYM/Mono. Higher LMR values are associated with lower CRC risk (protective effect); therefore, its OR is < 1.

- For all other biomarkers (OR > 1), higher values are associated with increased CRC risk.

Abbreviations: mSEPT9, methylated septin 9; DiAcSpm, N^1,N^{12}-diacetylspermine; NLR, neutrophil-to-lymphocyte ratio; PLR, platelet-to-lymphocyte ratio; LMR, lymphocyte-to-monocyte ratio; CEA, carcinoembryonic antigen; CA19-9, carbohydrate antigen 19-9; CA125, carbohydrate antigen 125; AFP, alpha-fetoprotein; CRC, colorectal cancer. Significance levels: ***p < 0.001; **p < 0.01; *p < 0.05.
