# Supplementary material for: Hypoxia Associated Integration of Epigenetic, Metabolic, and Immune Biomarkers in Blood and Urine for Early Colorectal Cancer Detection: A Multimarker Panel
Source: Diagnostics (Basel). 2026 Jun 6;16(12):1753. doi: 10.3390/diagnostics16121753 (PMC13298955; doi:10.3390/diagnostics16121753)
Supplement: Supplementary file 1 [file diagnostics-16-01753-s001.zip › Supplementary_Methods_file_1_(mSEPT9).pdf]

Supplementary Methods: Detailed assay procedure for mSEPT9 Quantification.

1. Methylated SEPT9 Detection

Cell-free DNA (cfDNA) was extracted from ≥4 mL plasma using a magnetic bead–based kit (Namagene, Wuhan, China). After bisulfite conversion was performed using a commercial kit (Biyuntian, Shanghai, China), methylation of the SEPT9 promoter was quantified by real-time quantitative methylation-specific PCR (QM-PCR) on a Bori platform. Primers and dual-labeled probes targeting methylated (FAM) and unmethylated (HEX) alleles were applied, with ACTB as an internal control. Methylation percentage was calculated from Ct differences between methylated and unmethylated alleles, calibrated against standard mixtures of methylated/unmethylated DNA.

Supplementary Methods – Methylated SEPT9 Assay

- **Thermal cycling conditions:**
  - Initial denaturation: 95 °C for 10 min
  - 40 cycles: 95 °C 15 s, 53 °C 30 s, 60 °C 30 s (fluorescence acquisition)
  - Hold: 4 °C
- **Primer/probe sequences and fluorophores (Supplementary Method Table S1):**

Target	Forward Primer (5′→3′)	Reverse Primer (5′→3′)	Probe	Fluorophore
mSEPT9 (methylated)	AAATAATCCCATCCAACTA	GTTGTTTATTAGTTATTATGT	ttaaccgcgaaatccgac	FAM-BHQ1
mSEPT9 (unmethylated)	AAATAATCCCATCCAACTA	GTTGTTTATTAGTTATTATGT	acattaaccacaaaatccaac	HEX-BHQ1
ACTB (internal control)	…	…	…	FAM/HEX

- **Ct cutoff rules:**
  - Exclude if ACTB Ct > 33 in triplex reaction
  - Exclude if both methylated and unmethylated Ct > 33

Methylation of SEPT9 in plasma DNA was measured using methylation-specific quantitative PCR (qMSP). Two approaches were initially evaluated to quantify methylation:

ΔCT-based methylation score: calculated as

- Methylation score (%)=100 / (1 + 2^ΔCT)

where ΔCT represents the difference between the CT of Methylated SEPT9 and the unmethylated SEPT9

2. **Calibration curve method:** methylation levels were also derived from a standard curve using serial dilutions of fully methylated control DNA.
Calibration curve derivation (Supplementary Method Figure S1):

- ○ $\Delta Ct = Ct_m - Ct_u$
- ○ Equation: $\Delta CT = -1.3485 - 1.4405 \log_2[c/(1-c)]$
- ○ c = methylation fraction (%)

- Where Ctm(FAM) and Ctu(HEX) reflect methylated/unmethylated alleles. This yielded a normalized % methylation (0–100%), validated against a calibration curve ($\Delta CT=-1.3485-1.4405\log2[c/(1-c)]$; $\Delta CT=Ctm-Ctu$; c= methylation fraction).
- Higher scores indicate greater SEPT9 promoter methylation, an epigenetic signature of **HIF-1α-driven gene silencing** in hypoxic tumor microenvironments.
- After preliminary comparison, the ΔCT-based methylation score was chosen for the final analyses, as it provided robust, reproducible, and comparable results across all samples. Calibration curve results were used only for method validation and quality control.

Supplementary Method Figure: Calibration curve for quantification of methylated SEPT9 by qPCR

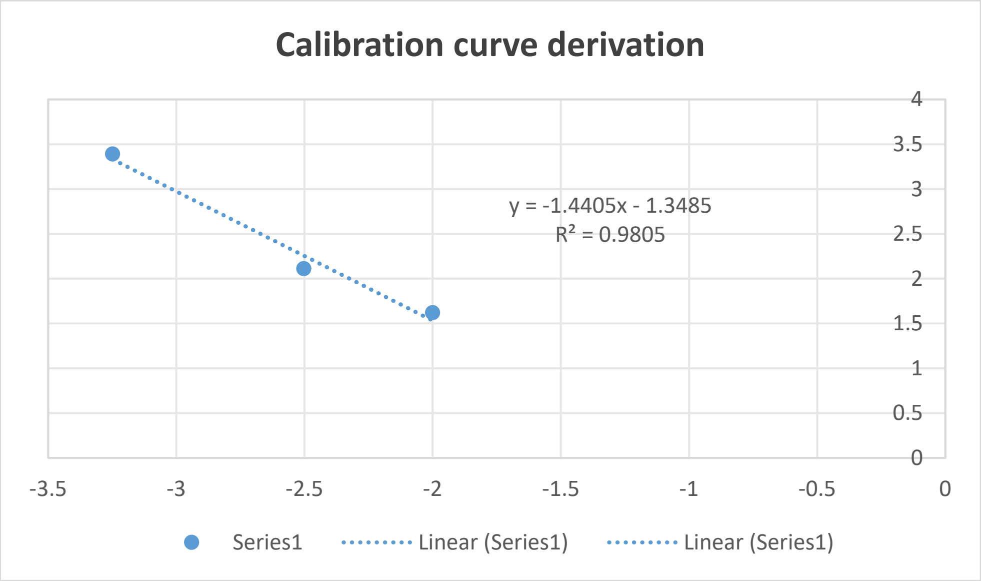

The calibration curve was generated using serial dilutions of methylated DNA standards and used to calculate mSEPT9 methylation percentages in plasma samples based on Ct values. The relationship between Ct and log (methylated DNA concentration) was determined by linear regression, and assay efficiency and correlation coefficients (R^2) were derived from the regression parameters.
