# Supplementary material for: Hypoxia Associated Integration of Epigenetic, Metabolic, and Immune Biomarkers in Blood and Urine for Early Colorectal Cancer Detection: A Multimarker Panel
Source: Diagnostics (Basel). 2026 Jun 6;16(12):1753. doi: 10.3390/diagnostics16121753 (PMC13298955; doi:10.3390/diagnostics16121753)
Supplement: Supplementary file 1 [file diagnostics-16-01753-s001.zip › Supplementary_Methods_File_2(DiAcSpm).pdf]

Supplementary Methods: Detailed assay procedure for Urinary N^1, N^{12}-Diacetylspermine Quantification.

Urinary N^1,N^{12}-Diacetylspermine (DiAcSpm) Quantification

Urinary N^1,N^{12}-Diacetylspermine (DiAcSpm) levels were quantified using a competitive ELISA kit (Shanghai Lianzu Biotechnology Co., Ltd., China) according to the manufacturer's instructions. Briefly, urine samples were diluted 1:5 in sample buffer before assay. Standards were prepared at concentrations of 0, 2.5, 5, 10, 20, and 40 ng/mL to generate a standard curve covering the expected detection range. Absorbance values were read at 450 nm using a microplate reader. Each 96-well plate contained a complete standard curve and internal quality control (QC) samples. All samples were assayed in duplicate. Inter-batch variation was minimized by running identical reagents and instrument settings across all plates within a batch. For each assay plate, the standard curve was fitted using an exponential regression model of the form:

$$y=a\times e^{bx}$$

where *y* is the predicted optical density (OD), *x* is the $\log_{10}$-transformed concentration, and *a* and *b* are plate-specific constants.

For this study, three independent ELISA plates (Excel Sheets 1–3) from "Data Sheet 2 raw data for DiAcSpm" were used, each with its respective calibration parameters: **Supplementary Method S2**

Plate	a	b	OD_0 (S0)	Notes
Sheet 1	1.7325	−2.4001	2.7619	138 samples
Sheet 2	1.6485	−2.3740	2.8240	138 samples
Sheet 3	1.7461	−2.3894	2.6740	106 samples

The final DiAcSpm concentrations (ng/mL) were calculated from the corresponding optical density (OD) values using the standard curve equation and multiplied by the dilution factor (×5). Data consistency across plates was verified using overlapping QC samples. Intra- and inter-assay coefficients of variation (CVs) were below 10%.

Standard curve fitting for urinary DiAcSpm quantification.

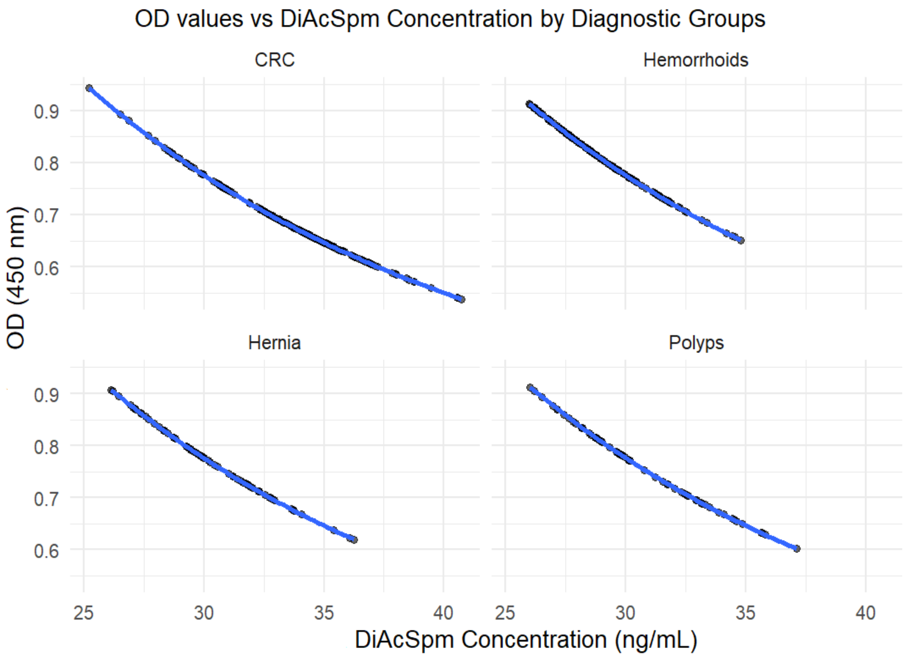

Representative calibration curves showing the relationship between optical density (450 nm) and DiAcSpm concentrations for each diagnostic group. A four-parameter logistic (4-PL) fit was applied, with $R^2 > 0.99$ for all assays.

Supplementary Method Figure S2. Distribution of urinary DiAcSpm concentrations across diagnostic groups.

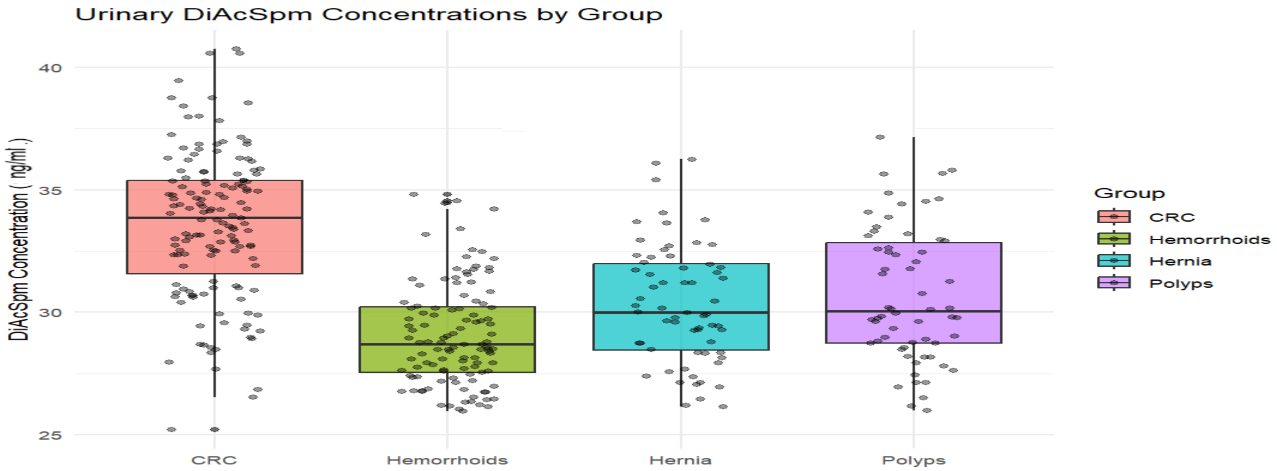

Boxplots represent median and interquartile range (IQR) values for colorectal cancer (CRC), polyps, and non-cancerous control groups. Outliers are shown as individual points.
