# Supplementary material for: Hypoxia Associated Integration of Epigenetic, Metabolic, and Immune Biomarkers in Blood and Urine for Early Colorectal Cancer Detection: A Multimarker Panel
Source: Diagnostics (Basel). 2026 Jun 6;16(12):1753. doi: 10.3390/diagnostics16121753 (PMC13298955; doi:10.3390/diagnostics16121753)
Supplement: Supplementary file 1 [file diagnostics-16-01753-s001.zip › Supplementary_Table_S12.pdf]

Supplementary Table S12. Sensitivity analyses of the D4 panel using different comparator groups.

Analysis	n (CRC / comparator)	AUC (95% CI)	Optimal cutoff	Sensitivity (%)	Specificity (%)
**Primary** (CRC vs. all non-CRC: polyps + hernia + hemorrhoids)	142 / 240	0.947 (0.924–0.970)	≥10.01% (mSEPT9) ≥32.32 ng/mL (DiAcSpm)	85.9	92.9
**Sensitivity 1** (CRC vs. colonoscopy-negative controls only)	142 / 42	0.971 (0.951–0.992)	– (same as primary)	91.5	92.9
**Sensitivity 2** (CRC vs. adenomatous polyps only, excluding hyperplastic polyps)	142 / 56	0.926 (0.884–0.968)	≥0.585*	92.3	85.7

Table S12.Performance of the D4 multimarker panel (mSEPT9 + DiAcSpm + NLR + PLR + LMR) under three different comparator definitions.

Groups:

- Primary analysis: CRC (n = 142) vs. all non-CRC (colorectal polyps, n = 62, plus hernia/hemorrhoid controls, n = 178; total non-CRC = 240).

- Sensitivity 1: CRC (n = 142) vs. only controls with negative colonoscopy (n = 42).

- Sensitivity 2: CRC (n = 142) vs. only adenomatous polyps (tubular, tubulovillous, villous, and serrated lesions; n = 56), excluding the six non-neoplastic hyperplastic polyps.

Metrics reported:

- n (CRC / comparator): sample sizes.

- AUC (95% CI): area under the ROC curve with confidence interval.

- Optimal cutoff: threshold used for classification. For Sensitivity 2, the cutoff (≥0.585) was derived from the Youden index applied to the logistic regression model on that subset (predicted probability scale); it is not directly comparable to the primary cutoffs (which are on the original biomarker scales: ≥10.01% for mSEPT9, ≥32.32 ng/mL for DiAcSpm).

- Sensitivity (%) and Specificity (%): at the respective optimal cutoffs.

Abbreviations: CRC, colorectal cancer; mSEPT9, methylated septin 9; DiAcSpm, N^1,N^{12}-diacetylspermine; NLR, neutrophil-to-lymphocyte ratio; PLR, platelet-to-lymphocyte ratio; LMR, lymphocyte-to-monocyte ratio; AUC, area under the curve; CI, confidence interval.

Interpretation:

- Sensitivity 1 (colonoscopy-negative controls) gives a higher AUC (0.971) than the primary analysis (0.947), indicating that verification bias did not inflate the results.

- Sensitivity 2 (adenomatous polyps only) yields an AUC of 0.926, close to the primary AUC (0.947) with overlapping confidence intervals, confirming that inclusion of the six hyperplastic polyps did not materially dilute the biomarker signal for neoplastic progression. Significance levels:** Not applicable (descriptive comparison only).
