# Supplementary material for: Hypoxia Associated Integration of Epigenetic, Metabolic, and Immune Biomarkers in Blood and Urine for Early Colorectal Cancer Detection: A Multimarker Panel
Source: Diagnostics (Basel). 2026 Jun 6;16(12):1753. doi: 10.3390/diagnostics16121753 (PMC13298955; doi:10.3390/diagnostics16121753)
Supplement: Supplementary file 1 [file diagnostics-16-01753-s001.zip › Supplementary_Table_S13.pdf]

Table S13. Comparison of unadjusted, baseline (age + sex only), and age-/sex-adjusted performance of the D4 multimarker panel.

Model	AUC (95% CI)	Sensitivity (%)	Specificity (%)
Unadjusted D4 panel	0.947 (0.924–0.970)	85.9	92.9
Baseline (Age + Sex only)	0.809 (0.765–0.853)	74.6	71.7
Adjusted D4 (Age + Sex)	0.962 (0.946–0.979)	94.4	86.2

Table S13. Performance of the D4 multimarker panel (mSEPT9 + DiAcSpm + NLR + PLR + LMR) before and after adjustment for age and sex, compared with a baseline model containing only age and sex.

Models:

Unadjusted D4 panel: original model without demographic covariates.

Baseline (Age + Sex only): logistic regression model using only age (continuous) and sex as predictors.

Adjusted D4 (Age + Sex): D4 panel plus age and sex as additional covariates.

Metrics reported:

AUC (95% CI): area under the ROC curve with confidence interval.

Sensitivity (%) and Specificity (%): at the Youden index threshold for each model.

Interpretation:

The baseline model (age + sex only) achieved an AUC of 0.809, demonstrating that demographic factors alone have moderate discriminatory ability.

The adjusted D4 panel significantly outperformed the baseline model (DeLong test $p < 0.0001$), confirming that the biomarkers provide substantial incremental diagnostic value beyond age and sex.

The age-/sex-adjusted D4 panel (AUC 0.962) also outperformed the unadjusted D4 panel (AUC 0.947), indicating that controlling for demographic factors further improves model performance.
