# Supplementary material for: Hypoxia Associated Integration of Epigenetic, Metabolic, and Immune Biomarkers in Blood and Urine for Early Colorectal Cancer Detection: A Multimarker Panel
Source: Diagnostics (Basel). 2026 Jun 6;16(12):1753. doi: 10.3390/diagnostics16121753 (PMC13298955; doi:10.3390/diagnostics16121753)
Supplement: Supplementary file 1 [file diagnostics-16-01753-s001.zip › Supplementary_Table_S14.pdf]

Table S14. Age- and sex-adjusted performance of the six combined biomarker models, with comparison to a baseline model (Age+Sex only).

Model	Adjusted AUC (95% CI)†	Adjusted Sensitivity (%)	Adjusted Specificity (%)	Baseline AUC (Age+Sex)	p value‡
A6	0.959 (0.939–0.979)	91.5	87.5	0.809 (0.765–0.853)	<0.0001
B8	0.925 (0.898–0.952)	95.8	74.6	0.809 (0.765–0.853)	<0.0001
C7	0.952 (0.930–0.974)	85.9	92.9	0.809 (0.765–0.853)	<0.0001
D1	0.965 (0.948–0.982)	93.0	87.5	0.809 (0.765–0.853)	<0.0001
D3	0.965 (0.948–0.982)	90.8	88.8	0.809 (0.765–0.853)	<0.0001
D4	0.962 (0.946–0.979)	94.4	86.2	0.809 (0.765–0.853)	<0.0001

Tale S14. Performance of six multimarker models after adjustment for age (continuous) and sex, compared with a baseline logistic regression model containing only age and sex. All analyses were performed on the complete case dataset (n = 382, excluding rows with missing values).
Models:

A6: mSEPT9 + DiAcSpm + PLR + NLR

B8: NLR + PLR + LMR + CEA + CA19-9

C7: CEA + CA19-9 + mSEPT9 + DiAcSpm + NLR

D1: mSEPT9 + DiAcSpm + NLR + PLR + LMR + CEA + CA19-9

D3: mSEPT9 + DiAcSpm + NLR + PLR + CEA + CA19-9 + CA125 + AFP

D4: mSEPT9 + DiAcSpm + NLR + PLR + LMR

Metrics reported:

Adjusted AUC (95% CI): area under the ROC curve after including age and sex as covariates.

Adjusted Sensitivity (%) and Adjusted Specificity (%): at the Youden-optimised threshold.

Baseline AUC (Age + Sex): AUC of the model containing only age and sex (0.809; 95% CI 0.765–0.853).

p value‡: DeLong test comparing the adjusted model to the baseline model.

Baseline model (Age + Sex alone) had a sensitivity of 74.6% and specificity of 71.7%.
All adjusted models significantly outperformed the baseline model ($p < 0.0001$), confirming that the biomarkers provide substantial diagnostic value beyond demographic factors alone.
