# Supplementary material for: Hypoxia Associated Integration of Epigenetic, Metabolic, and Immune Biomarkers in Blood and Urine for Early Colorectal Cancer Detection: A Multimarker Panel
Source: Diagnostics (Basel). 2026 Jun 6;16(12):1753. doi: 10.3390/diagnostics16121753 (PMC13298955; doi:10.3390/diagnostics16121753)
Supplement: Supplementary file 1 [file diagnostics-16-01753-s001.zip › Supplementary_Table_S15.pdf]

Table S15. Performance of the D4 multimarker panel after propensity score matching (1:1 matching on age and exact matching on sex).

Analysis	n (CRC / controls)	AUC (95% CI)	Sensitivity (%)	Specificity (%)
Primary (unadjusted, all non-CRC)*	142 / 240	0.947 (0.924–0.970)	85.9	92.9
Age + sex adjusted (covariate)	142 / 240	0.962 (0.946–0.979)	94.4	86.2
Propensity score matched	78 / 78	0.967 (0.943–0.990)	87.2	94.9

Table S15: Comparison of the D4 panel's diagnostic performance under three analytical approaches: primary (unadjusted), covariate adjustment, and propensity score matching. All analyses used the complete case dataset (n = 382).

Analyses:

Primary (unadjusted, all non-CRC): CRC (n = 142) vs. all non-CRC (polyps + hernia/hemorrhoid controls, n = 240); no adjustment for age or sex.

Age + sex adjusted (covariate): same dataset but with age (continuous) and sex added as covariates in the logistic regression model.

Propensity score matched: 1:1 nearest-neighbour matching on age (caliper = 0.2 on the propensity score) and exact matching on sex; retained 78 CRC patients and 78 controls with balanced age (mean 59.8 vs. 59.0 years) and identical sex distribution.

Metrics reported:

n (CRC / controls): sample sizes.

AUC (95% CI): area under the ROC curve with confidence interval.

Sensitivity (%) and Specificity (%): at the Youden-optimised threshold for each analysis.

Interpretation:

The propensity-score-matched analysis yielded an AUC of 0.967 (95% CI 0.943–0.990), sensitivity 87.2%, and specificity 94.9%, which is consistent with the age-/sex-adjusted covariate analysis (AUC 0.962).

These results confirm that the diagnostic performance of the D4 panel is not driven by age or sex imbalances but reflects genuine tumour-associated signals.
