# Supplementary material for: Hypoxia Associated Integration of Epigenetic, Metabolic, and Immune Biomarkers in Blood and Urine for Early Colorectal Cancer Detection: A Multimarker Panel
Source: Diagnostics (Basel). 2026 Jun 6;16(12):1753. doi: 10.3390/diagnostics16121753 (PMC13298955; doi:10.3390/diagnostics16121753)
Supplement: Supplementary file 1 [file diagnostics-16-01753-s001.zip › Supplementary_Table_S16.pdf]

Table S16. Age- and sex-adjusted multivariable logistic regression odds ratios for colorectal cancer detection (all nine biomarkers included simultaneously).

Biomarker	OR (95% CI)	Z-score (ORs per 1 SD increase)	p-value
mSEPT9	1.14 (1.07–1.21)	3.97	<0.001 ***
DiAcSpm	1.47 (1.26–1.71)	4.96	<0.001 ***
NLR	2.64 (1.71–4.09)	4.36	<0.001 ***
PLR	1.01 (1.00–1.02)	2.53	0.012 *
LMR	0.65 (0.46–0.91)	2.54	0.011 *
CEA	1.02 (1.00–1.04)	1.57	0.117
CA199	1.01 (1.00–1.02)	2.43	0.015 *
CA125	1.02 (0.97–1.06)	0.71	0.481
AFP	1.07 (1.00–1.15)	1.90	0.057

Table S16: Results from a multivariable logistic regression model that included all nine biomarkers (mSEPT9, DiAcSpm, NLR, PLR, LMR, CEA, CA19-9, CA125, AFP) plus age (continuous) and sex as covariates. Analysis based on 382 complete cases (non-CRC group includes colorectal polyps, n=62, and non-malignant controls, n=178; total non-CRC = 240).
Metrics reported:

OR (95% CI): odds ratio per **one standard deviation increase** in each biomarker (standardised Z-scores), allowing direct comparison of effect sizes across biomarkers measured on different scales.

Z-score: Wald statistic (coefficient divided by its standard error) for the standardised model.

p value: two-tailed significance level.
Interpretation:

LMR (lymphocyte-to-monocyte ratio) is defined as LYM/Mono; higher LMR values are associated with lower colorectal cancer risk (protective effect), hence its OR is < 1.

For all other biomarkers (OR > 1), higher values are associated with increased CRC risk.
Significance levels: *** p < 0.001; ** p < 0.01; * p < 0.05.
