# Supplementary material for: Hypoxia Associated Integration of Epigenetic, Metabolic, and Immune Biomarkers in Blood and Urine for Early Colorectal Cancer Detection: A Multimarker Panel
Source: Diagnostics (Basel). 2026 Jun 6;16(12):1753. doi: 10.3390/diagnostics16121753 (PMC13298955; doi:10.3390/diagnostics16121753)
Supplement: Supplementary file 1 [file diagnostics-16-01753-s001.zip › Supplementary_Table_S17.pdf]

Supplementary Table S17

Table S17. Logistic regression coefficients for the D4 multimarker panel (raw units).

Predictor	Coefficient (B)	Standard Error	z	p-value
Intercept	–17.127	2.552	–6.71	<0.001
mSEPT9	0.130	0.027	4.74	<0.001
DiAcSpm	0.382	0.070	5.45	<0.001
NLR	1.057	0.200	5.28	<0.001
PLR	0.012	0.003	3.79	<0.001
LMR	–0.467	0.150	–3.11	0.002

Table S17. Logistic regression coefficients for the final D4 multimarker panel (mSEPT9 + DiAcSpm + NLR + PLR + LMR) fitted on the complete case dataset (n = 382).
Metrics reported:

Coefficient (B): raw (unstandardised) regression coefficient (log-odds scale).

Standard Error: standard error of the coefficient.

z: Wald z-statistic (coefficient divided by its standard error).

p value: two-tailed significance level.

LMR: lymphocyte-to-monocyte ratio (LYM/Mono). The negative coefficient for LMR indicates a protective effect (higher LMR → lower CRC risk).

Predicted probability formula:

Where

$P=1/1+e^{-logit}$

$logit=-17.127+0.130\times mSEPT9+0.382\times DiAcSpm+1.057\times NLR+0.012\times PLR-0.467\times LMR$

All predictors are entered as raw values (no scaling). The model was fitted on the complete case dataset (n = 382).
